# Development and validation of a high-density ‘Amahysnp’ genotyping array in grain amaranth (*Amaranthus hypochondriacus*)

**DOI:** 10.1186/s12870-025-07367-z

**Published:** 2025-10-01

**Authors:** Rakesh Singh, Ajay Kumar Mahato, S. Rajkumar, A.K. Singh, Akshay Singh, Avantika Maurya, Rajat Gupta, Rakesh Bhardwaj, S. K. Kaushik, Sandeep Kumar, Veena Gupta, Kuldeep Singh, G. P. Singh

**Affiliations:** 1 Division of Genomic Resources, ICAR-National Bureau of Plant Genetic Resources, Pusa Campus, New Delhi, India; 2 Centre for DNA Fingerprinting and Diagnostics, Hyderabad, India; 3 Division of Germplasm Evaluation, ICAR- National Bureau of Plant Genetic Resources, Pusa Campus, New Delhi, India; 4 Division of Germplasm Conservation, ICAR- National Bureau of Plant Genetic Resources, Pusa Campus, New Delhi, India; 5 International Crops Research Institute for the Semi-Arid Tropics, Patancheru, Telangana India; 6 ICAR-National Bureau of Plant Genetic Resources, Pusa Campus, New Delhi, India

**Keywords:** Single nucleotide polymorphism (SNPs), SNP chip array, Genetic diversity, QTNs, Candidate genes, GWAS

## Abstract

**Background:**

Grain amaranth has recently gained global attention as a promising crop alternative to traditional cereals due to its nutritional value and adaptability to various growing conditions. Although gene banks conserve extensive collections of amaranth germplasm, the genomic and phenotypic characterization of these resources is limited, which hinders their full utilization in breeding programs. A major challenge is the lack of high-throughput genotyping assays essential for comprehensive genomic characterization and trait mapping. High-density SNP arrays have become standard tools for genome-wide analysis across multiple loci, enabling molecular breeding across a range of crop species.

**Results:**

In this study, we developed a 64 K high-throughput SNP genotyping array named “AmahySNP”, using Affymetrix^®^ Axiom^®^ technology. The array contains 64,069 high-density SNPs distributed across both genic (55.17%) and non-genic (44.83%) regions of the *Amaranthus hypochondriacus* genome. The genic region includes 8,879 genes, which consist of 4,830 single-copy genes and 4,049 multi-copy genes distributed across 16 scaffolds. These genes cover various functional regions, including exons (10.5%), introns (40.1%), 5’UTRs (1.6%), and 3’UTRs (2.9%), respectively. The AmahySNP array was effectively utilized for population structure analysis, genetic diversity studies, core development, and genome wide association studies (GWAS) in amaranth germplasm. A representative core set of 112 accessions was identified, which includes two released varieties (Annapurna and Suvarna) and 100 diverse accessions from 12 different regions, representing 12% of the total 917 accessions evaluated. Phylogenetic analysis revealed three major genetic clusters, independent of their geographical origins. GWAS conducted using 22,763 polymorphic SNPs from 540 genotypes identified 13 novel loci associated days to flowering (DTF) trait, seven of which were located within annotated genes.

**Conclusions:**

The AmahySNP 64 K SNP chip a valuable genomic tool for amaranth research and breeding with a strong potential to accelerate its genetic improvement. It enables high-throughput genotyping for a wide range of applications, including GWAS and other genomic studies, and will significantly advance the exploration of natural genetic variations. Ultimately, this resource will empower amaranth breeders to develop improved amaranth cultivars with enhanced crop yield, resilience, and nutritional quality, contributing to global food security and sustainable agriculture.

**Supplementary Information:**

The online version contains supplementary material available at 10.1186/s12870-025-07367-z.

## Background

Grain amaranth (*Amaranthus spp.*), a C4 dicotyledonous pseudocereal, is a highly nutritious source of protein, vitamins, and minerals, with great potential for addressing global food security. The name *Amaranthus* is derived from the Greek word “anthos” (flower), meaning everlasting or eternal [1]. The genus *Amaranthus* comprises 70–75 species, primarily originating in the Americas, and is categorized based on their usage into grain amaranth, vegetable amaranth, ornamental types, and weedy species [2, 3]. The grain amaranth group includes species such as *Amaranthus hypochondriacus*, *Amaranthus cruentus*,* Amaranthus caudatus*, and *Amaranthus edulis* [4]. Due to their nutraceutical potential, agronomic versatility, and potential to address the serious global issue of food security, it is often referred to as the “golden crop for the future” [5]. Amaranth is considered an emerging superfood because it is a gluten-free grain source for celiac disease patients and comprises all the components of a balanced diet, such as iron (Fe), calcium (Ca), magnesium (Mg), dietary fibers, unsaturated oils, flavonoids, vitamins A, C, and E, and a high source of protein [6, 7]. Its high nutrient content makes it an excellent addition to the diet, providing a range of health benefits and the potential to alleviate malnutrition and food poverty in various communities [8]. Grain amaranth has higher oil (7.8–11.6%) and protein (13.2–18.2%) content compared to other pseudo cereals. Its oil, rich in tocopherols and phytosterols, promotes heart health and may reduce risks of coronary heart disease, prostate cancer, osteoporosis, and anaemia, while supporting the immune system [9, 10].

Amaranth is highly adaptable to diverse environments, including heat and drought, and thrives in arid and semi-arid regions. It has a short growing cycle (8–12 weeks) and produces higher yields than maize and rice with lower inputs such as water and fertilizer, making it suitable for cultivation on a variety of soils [11, 12]. India, particularly the Himalayan region, is a major producer of amaranth; however, its productivity remains low due to limited yields and poorly characterized germplasm collections [13]. The development of high-yielding and nutrient-rich amaranth cultivars is hindered by a lack of genome-level characterization of germplasm and an insufficient understanding of the genetic mechanisms regulating key traits [14]. Genotyping with molecular markers is essential for the genetic characterization of amaranth germplasm, assessment of genetic diversity, identification of redundant accessions in gene banks, development of molecular core collections, quantitative trait loci (QTL) mapping, and marker-assisted selection [15, 16]. Several studies have employed molecular markers such as random amplified polymorphic DNAs (RAPDs) [17–19], restriction fragment length polymorphisms (RFLPs) [20], amplified fragment length polymorphisms (AFLPs) [21, 22], and simple sequence repeats (SSRs) [23, 24] in grain amaranth. However, these low-density PCR-based markers often fail to maintain linkage with trait loci [25]. Therefore, the development of evenly distributed, high-throughput single nucleotide polymorphism (SNP) markers is crucial to advance amaranth genetics and breeding.

High-throughput genotyping assays and genotyping-by-sequencing (GBS) are key platforms for large-scale SNP discovery [15]. Compared to GBS and re-sequencing, high-density arrays offer more consistent data output and simplified downstream analysis [26]. Several high-density arrays have been developed for various crops, including the rice 50 K genic chip (OsSNPnks) [27], the rice pangenome genotyping array (RPGA) [28], pigeon pea (CcSNPnks) [29], CottonSNP80K array [30], maize [31], and sunflower [32], as well as for animal species like cattle (BovineSNP50) [33] and pigs (PorcineSNP60) [34]. To date, only a few studies have reported genome-wide SNP marker development and application in amaranth [35, 36]. In earlier research, Mahagun (2009) [35], identified 27,658 SNPs via genomic reduction, barcoding, and 454-pyrosequencing in an amaranth population. Another study identified 10,668 SNPs in 94 amaranth accessions using the GBS approach [37]. However, these GBS-derived SNPs are unevenly distributed across the amaranth genome, as genomic DNA fragments in a GBS library are randomly represented, and SNP detection depends on the sequence coverage across selected genotypes. Moreover, the applicability of GBS-based SNPs is limited to the genotypes used during their discovery, limiting their utility in broader genetic studies. To overcome these limitations, the present study focused on developing a high-density SNP array for amaranth to support diversity studies, genetic mapping, core collection development, association mapping, and molecular breeding.

We report the development and application of the Amaranth SNP array on the Affymetrix GeneTitan^®^ platform, which contains 64,000 SNP markers and is named ‘AmahySNP’ (*Amaranthus hypochondriacus* single nucleotide polymorphism). The SNPs for the array were identified by analyzing resequencing data from a diverse set of amaranth genotypes aligned against the high-quality reference genome of *Amaranthus hypochondriacus*. The utility of this SNP chip has been demonstrated through genetic diversity analysis, population structure studies, core collection development, and genome-wide association studies (GWAS) focused on traits such as days to flowering to identify associated loci.

## Methods

### Plant materials

A total of 917 genetically diverse grain amaranth accessions were selected based on seed availability and passport information and obtained from the National Gene Bank of India at the ICAR-National Bureau of Plant Genetic Resources (ICAR-NBPGR), New Delhi, representing 22 distinct geographic locations across various agro-climatic regions (Table S1). All 917 selected accessions were grown during the Kharif season of 2019–2020 and the Rabi season of 2020–2021 at ICAR-NBPGR, New Delhi, following the augmented block design. Each accession was shown in a single-row plot of 3 m in length, with 30 cm row-to-row and 10 cm plant-to-plant spacing. To ensure self-pollination and minimize outcrossing, the inflorescences were covered with three ring bags. Seeds were harvested from single plants to prevent mechanical mixing and to maintain genetic purity. For DNA isolation, seeds from these plants were sown in a greenhouse at ICAR-NBPGR, New Delhi, using hyco-trays (8 cm × 8 cm × 8 cm) under controlled temperatures ranging from 25 °C to 40 °C. Fresh, tender leaves were harvested, flash-frozen in liquid nitrogen, and stored at −80 °C until DNA extraction.

### SNP identification and array development

To identify genome-wide SNPs, raw genome sequencing data of amaranth accessions from India (SRX1118959, 15.4 Gb), Nepal (SRX1120735, 15.4 Gb), Pakistan (SRX1120675, 15.2 Gb), and Mexico (SRX1119734, 15.2 Gb), available in the NCBI SRA database, were analyzed. The high-quality grain amaranth reference genome published by Lightfoot et al. (2017) [38] was used for SNP identification. SNP mining was performed using the standard Genome Analysis Toolkit (GATK) workflow [39], which resulted in the identification of approximately 850,000 SNPs. High-quality SNPs were filtered based on read coverage, mapping quality, and in silico validation using the Affymetrix Power Tool (APT) AxiomGTv1. Probes (35-nucleotide sequences flanking each SNP) with *p*-convert values greater than 0.50 were retained, indicating a high probability of successful assay conversion, while probes below this threshold were excluded. SNPs were further categorized as ‘neutral,’ ‘recommended,’ or ‘not recommended’ to streamline the filtration process. Ultimately, 64,069 high-quality SNPs were selected for the construction of the Amaranth Affymetrix SNP chip.

### Array hybridization and genotyping with 64 K SNP array

Genomic DNA was isolated from plant leave tissues using a multi-step protocol described by Sahu et al. 2019 [40], with certain modifications. DNA quality was assessed using a Nanodrop 1000 spectrophotometer (Thermo Scientific, USA), ensuring a 260/280 ratio in the range of 1.7–1.9 and a 260/230 ratio between 1.5 and 2.2. For target probe preparation, 20 µl of genomic DNA at a concentration of 20 ng/µl was used, following the guidelines provided in the Affymetrix Axiom 2.0 Assay Manual. The protocol included DNA denaturation, neutralization, amplification, precipitation, washing with isopropyl alcohol, and fragmentation. The DNA pellets were then dried, resuspended, and subjected to a 23-hour chip-based hybridization phase. This was followed by DNA ligation, signal amplification, washing, staining, and final scanning using the GeneTitan^®^ Multi-Channel Instrument, as per the manufacturer’s instructions.

### Data analysis and pruning of SNP alleles

The Affymetrix Genotyping Console software was used to extract data from the library files, while GCOS Affymetrix software was utilized for initial data generation. CEL files were converted into genotype calls using the Axiom™ Analysis Suite 2.0. High-performing SNPs with a dish quality control (DQC) value > 0.85 and call rates > 95% were selected. The dataset was then formatted for quality evaluation and analyzed using the Axiom™ Analysis Suite 2.0 workflows, including Sample QC, Genotyping, and Summary. Missing genotypes were imputed using Beagle 5.0, and SNP pruning was performed using PLINK v1.07. Only SNPs with a minor allele frequency (MAF) greater than 5% were retained and considered for further analysis.

### Functional annotation of single-nucleotide polymorphisms

The flanking sequences of quality-filtered SNPs were analyzed using Blast2GO [41]. BlastX searches were performed against a locally configured non-redundant (nr) protein database to identify putative gene functions. Redundant hits were removed, and Gene Ontology (GO) terms were assigned to each unique SNP using Blast2GO. The associated genes were classified into three broad GO categories: cellular components, biological processes, and molecular functions.

### Population structure, phylogenetic and molecular diversity analysis

A total of 917 Amaranthus genotypes, including released varieties, landraces, and elite Lines, were used for diversity analysis after passing genotyping quality checks. A total of 24,203 quality-filtered SNP markers were used to infer subpopulations using the Bayesian model-based software STRUCTURE v2.3.4 [42], with parallelization facilitated by StrAuto to reduce computational time [43]. K values ranging from 1 to 10 were tested, with three independent replicates for each K, using 100,000 burn-in and 100,000 Markov Chain Monte Carlo (MCMC) iterations. STRUCTURE HARVESTER was used to determine the optimal number of subpopulations (K) [44]. A neighbor-joining phylogenetic tree of 917 genotypes was constructed using pruned SNPs in TASSEL v5 [45] and visualized with iTOL v6 [46]. Principal component analysis (PCA) and genetic diversity indices, including Nei’s genetic diversity (GD), polymorphic information content (PIC), minor allele frequency (MAF), and heterozygosity, were calculated using the R packages ‘SNPRelate’ and ‘adegenet’ [47, 48].

### Development of core collection and diversity assessment

A core collection was developed using 24,203 quality-filtered SNPs with the ‘Core Hunter 3’ package [49], representing 12% of the total population. The core set was developed based on two optimization strategies: average entry-to-nearest-entry distance (Modified Rogers distance) and Shannon’s allelic diversity index (SH). To evaluate the core, various genetic diversity indices were calculated and compared with the entire population. The phylogenetic tree construction and PCA were performed using TASSEL v5 [45], while population structure analysis was performed using STRUCTURE software, with three iterations for each K value (1 to 10), using 100,000 burn-in and MCMC repetitions.

### GWAS analysis of the DTF trait

Phenotypic data for the days to flowering (DTF) trait were recorded for 540 out of 917 amaranth accessions during the 2019–2020 Kharif (E1) and 2020–2021 Rabi (E2) seasons and were used for GWAS analysis. Pairwise linkage disequilibrium (LD) was calculated using TASSEL v5 [45], with *r*^2^ values estimated for alleles within a sliding window of 50 SNPs. The LD decay rate was defined as the distance at which the *r*² value declined to half of its average maximum value, as a function of the chromosomal distance [50]. This was determined by plotting the *r*² values against the physical distances (in base pairs) between locus pairs. The LD decay value was determined by identifying the intersection point between the LD curve and the specified r² threshold. GWAS was performed on 540 amaranth accessions using a filtered set of 22,763 SNPs for both the Kharif and Rabi seasons. Single-locus GWAS (SL-GWAS) was conducted using the mixed linear model (MLM) and compressed MLM (CMLM) implemented in GAPIT 3.0 to reduce false positives [51]. To identify the candidate QTNs and validate the peaks identified by SL-GWAS, four multi-locus GWAS (ML-GWAS) models, mrMLM, FASTmrMLM, FASTmrEMMA, and pLARmEB were applied using the R package mrMLM (https://cran.r-project.org/web/packages/mrMLM/index.html [52],. For all models, a kinship matrix calculated in TASSEL v5 was used to account for relatedness among accessions. Significant QTNs were identified with a LOD score threshold of ≥ 3, as described by Duan et al. 2017 [53]. QTNs validated by at least two ML-GWAS methods and SL-GWAS methods were considered true positives.

###  Candidate gene identification

Genes located within 695 kb upstream or downstream of the peak-annotated SNPs were considered candidate genes for the DTF trait. Gene annotation was performed using Phytozome 13 (https://phytozome-next.jgi.doe.gov/info/Ahypochondriacus_v2_1*)* and the Amaranth Genomic Resource Database [54].

## Results

### Design and characterization of the Amaranth 64 K array

**Fig. 1:**
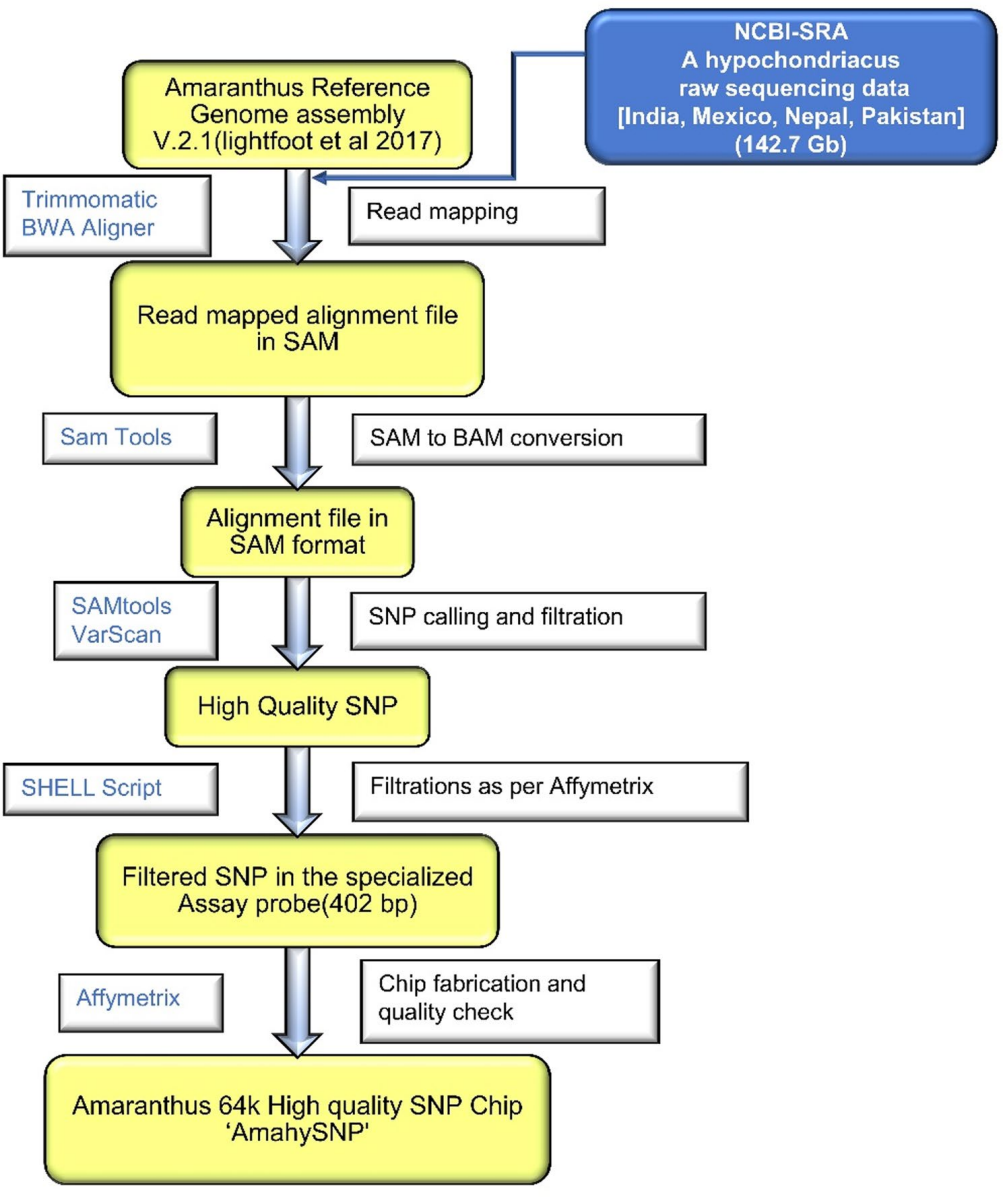
Flow diagram of 64 K SNP chip development and fabrication from nine different *A. hypochondriacus* resequencing data. Yellow color boxes represent the input and output in consecutive steps. The blue text box on the left side of the arrows represents the software used, and the black text boxes on the right side display the methods

A 64 K SNP array, named AmahySNP, was developed based on the high-quality *A. hypochondriacus* genome sequence (v2.1) and raw sequencing data from nine grain amaranth genotypes representing diverse geographic locations, including India, Nepal, Pakistan, and Mexico, using an in-house semi-automated pipeline (Fig. 1). The raw sequencing reads were retrieved from the NCBI-SRA database and mapped to the *A. hypochondriacus* reference genome using Bowtie software [55]. The Genome Analysis Toolkit (GATK) suite was employed to identify high-quality SNPs, which were selected based on read coverage and overall mapping quality. High-quality SNPs were filtered based on a *p*-value threshold of 0.99 and a minimum coverage of 50X, reducing 376 million raw SNPs to 999,542 high-quality SNPs. The VCF files were further processed to select SNPs that fulfilled the manufacturer’s criteria for SNP genotyping chip fabrication. A feature sequence of 402 bp was generated for each filtered SNP, resulting in the development of a high-density SNP genotyping chip comprising 64,069 probes representing 8,879 genes across 16 scaffolds.

**Fig. 2:**
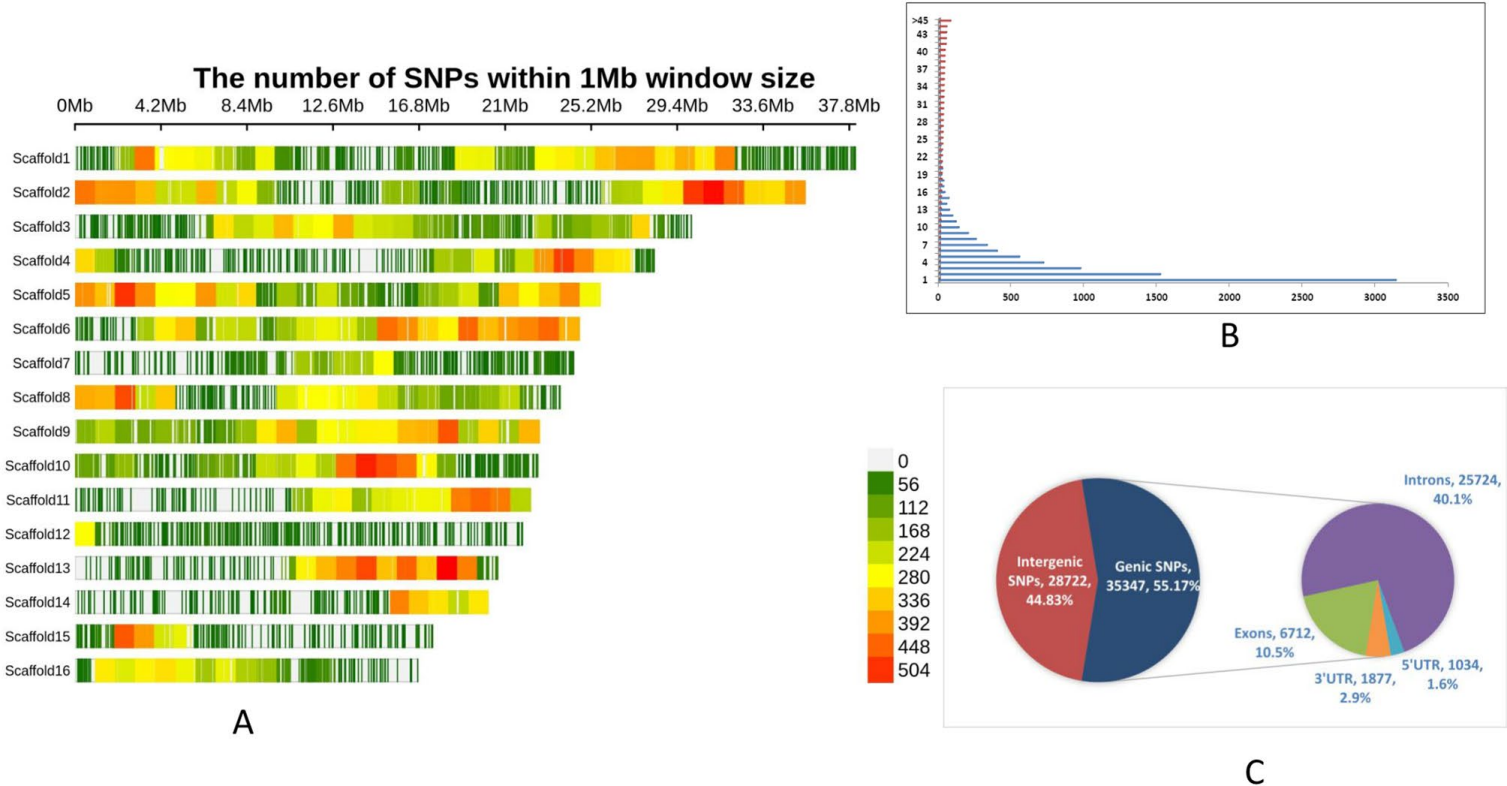
Distribution of 64,069 SNPs in the AmahySNP array. (**A**) SNP distribution within a 1 Mb window on the 16 scaffolds of *A. hypochondriacus.* (**B**) Frequency distribution of SNPs per gene in the 64k SNP chip incorporating 8879 genes. (**C**) Distribution of quality-filtered 64,609 SNPs in genic and intergenic regions

The AmahySNP array contained 64,069 non-redundant SNPs, of which 55.17% (35,347) were genic and 44.83% (28,722) were intergenic SNPs, distributed across 16 scaffolds. Among the genic SNPs, 10.5% (6,712) were located in exons, 40.1% (25,724) in introns, 1.6% (1,034) in 5’ UTRs, and 2.9% (1,877) in 3’ UTRs (Fig. 2). The SNPs were evenly distributed across all 16 scaffolds, with an average distance of 6.17 Kb between adjacent SNPs and an average of 3.98 SNPs per gene. Scaffold 6 exhibited the highest SNP density (~ 1 SNP per 3.8 Kb, with 6,451 SNPs), while scaffold 12 had the lowest density (~ 1 SNP per 32.3 Kb, with 683 SNPs) (Table S2).

In this study, the proportion of transition SNPs (31,625 allelic sites, 49.36%) was greater than that of transversion SNPs (20,768 allelic sites, 32.41%), resulting in a transition/transversion ratio of 1.52, indicating a biased mutational process. The most frequent transition variant was A: G (9,048 sites, 14.12%), while the least frequent transversion variant was C: G (1,806 sites, 2.82%). Detailed SNP statistics and allele frequency data for the 64 K chip are provided in Table S3.

**Fig. 3:**
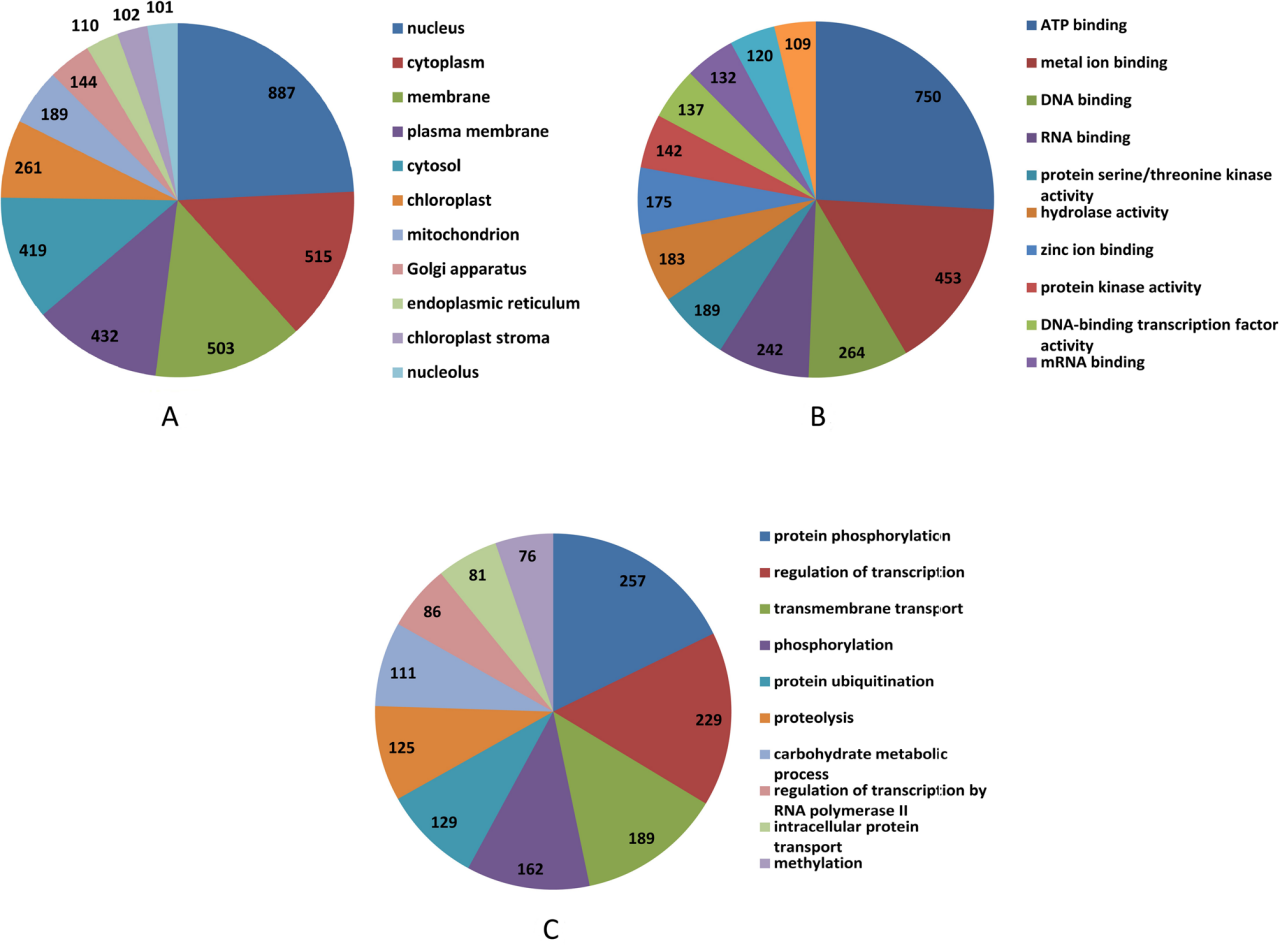
Gene ontology annotation of the 64 K SNPs. (**A**): GO of cellular components of 64k SNPs (**B**): GO of molecular functions of 64k SNPs (**C**): GO of biological processes of 64k SNPs

The SNP-containing genes were categorized into cellular components, molecular functions, and biological processes based on functional annotation data. Approximately 42% of the SNPs were associated with cellular components, including the nucleus (887 genes), cytoplasm (525 genes), membrane (503 genes), plasma membrane (432 genes), cytosol (419 genes), chloroplasts (261 genes), mitochondria (189 genes), and endoplasmic reticulum (110 genes). Genes with diverse molecular functions were identified, such as ATP binding (750 genes), metal ion binding (453 genes), DNA binding (264 genes), RNA binding (242 genes), protein serine/threonine kinase activity (189 genes), hydrolase activity (183 genes), zinc ion binding (175 genes), protein kinase activity (142 genes), and DNA-binding transcription factor activity (137 genes). In terms of biological processes, the genes were involved in protein phosphorylation (257 genes), regulation of transcription (229 genes), transmembrane transport (189 genes), phosphorylation (162 genes), protein ubiquitination (129 genes), proteolysis (125 genes), carbohydrate metabolism (111 genes), regulation of transcription by RNA polymerase II (86 genes), intracellular protein transport (81 genes), and methylation (76 genes) (Fig. 3). This functional classification of SNPs highlights the molecular roles of genes across a range of c cellular compartments and biological processes.

### Fabrication and validation of the 64 K SNP chip

The probe sequences for all 64,069 filtered SNPs were evaluated in silico using the Affymetrix Power Tool (APT) Axiom GTv1 algorithm to optimize probe selection for a high-quality SNP chip. The *p*-convert value, a predictive score generated by the APT software, estimates the probability of successful conversion of a SNP into a reliable genotyping assay on the Axiom^®^ platform. It incorporates multiple parameters such as probe binding specificity, thermodynamic properties, secondary structure potential, and sequence context. SNPs with *p*-convert values > 0.50 are considered high quality and likely to perform well during hybridization and allele calling. Therefore, only SNPs exceeding this threshold were retained for array fabrication to ensure robust and reproducible genotyping performance. Initially, 64,069 probes were tiled on the array with two repetitions per marker on the recommended strand. To utilize the remaining array space, complementary strands were tiled in the neutral and not recommended categories at two repetitions, thereby enhancing SNP/genotype detection. Finally, the complementary strand was tiled in reverse p-convert order using the “recommended” category to maximize space efficiency. In total, 70,346 probes were included, representing 64,069 SNPs, ensuring successful conversion of the intended Axiom assays into the final chip. The chip includes 4,810 single-copy genes and 4,049 multi-copy genes. Ultimately, 7.5% of the initial 850,000 SNPs were incorporated into the chip after rigorous filtration. Chip performance was experimentally validated by genotyping a set of 917 diverse *A. hypochondriacus* accessions using the Affymetrix Gene Titan^®^ platform. All samples passed DQC (> 0.85) and call rate (> 95%) thresholds, with an average QC call rate of 99.3%, confirming the chip’s high reliability.

### Population stratification and genetic diversity analysis

**Fig. 4:**
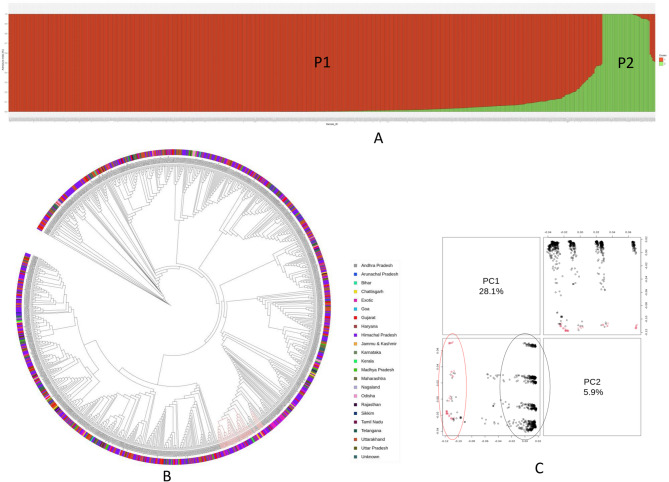
Genetic diversity and population structure analysis of 917 *A. hypochondriacus* accessions based on 24,203 pruned SNP markers detected using the AmahySNP assay. (**A**) Bar plot generated using model-based structure analysis showing the assignment of amaranth accessions into two subpopulations (P1 and P2). (**B**) Neighbour-joining phylogenetic tree. The outer color strip illustrates the different geographical locations of each accession, and the black and red branches represent the accessions from the P1 and P2 subpopulations, respectively and (**C**) Principal component analysis plot: different colors represent different groups

SNP pruning was performed to retain only high-quality and informative markers for analysis. SNPs with more than 10% missing data were excluded, followed by removal of SNPs with a MAF below 0.05. After filtering, 24,203 SNPs were retained, and 39,866 were excluded from further analysis. The genetic diversity and population structure of 917 *A. hypochondriacus* accessions were analyzed using the 24,203 pruned SNPs (MAF ≥ 0.05). The panel included an indigenous collection (IC) of 875 Indian accessions and an exotic collection (EC) of 42 accessions from various regions of the world. The optimal number of genetic clusters (K) was determined using a structure-based clustering method and Evanno’s ΔK’ approach, which calculates the rate of change in likelihood. The peak ΔK value occurred at K = 2, indicating the most probable number of populations in the study. Accessions with a probability score ≥ 80 were considered genetically pure, while those with a score ≤ 80 were classified as admixed. Subpopulation P1 (red) consisted of 874 accessions, 788 indigenous and 29 exotic pure accessions, along with 57 admixed accessions. Subpopulation P2 (green) comprised 43 accessions, of which 35 were pure and 8 were admixed, with 83.72% being indigenous and 16.28% exotic. Groupings were visualized in a bar plot generated using R (Fig. 4A).

Phylogenetic analysis using the neighbour-joining algorithm in the TASSEL v5 divided the 917 genotypes into three clusters: I, II, and III (Fig. 4B). Cluster I contained one accession, IC0038406, from Banera, Himachal Pradesh. Cluster II included three indigenous genotypes, IC0448747 from Uttarakhand, and IC0398237 and IC0035593 from Gujarat. Cluster III consisted of 913 accessions, including landraces, elite lines, and released varieties. Clusters I, II, and III corresponded to the P1 subpopulation from STRUCTURE (black branches), while a sub-cluster within Cluster III aligned with the P2 subpopulation (red branches) (Fig. 4B). The dendrogram indicated that SNPs did not group accessions strictly by geographic origin, as genotypes from different regions were interspersed across sub-clusters. The PCA analysis based on 24,203 SNPs explained 28.1% and 5.9% of the variance with the first and second principal components, respectively. PCA separated the accessions into two major clusters corresponding to the P1 and P2 subpopulations, with eight subclusters suggesting admixture across geographical regions (Fig. 4C).

Several genetic diversity indices were calculated for the 917 amaranth genotypes. Nei’s genetic diversity (GD) ranged from 0.1 to 0.5, with an average of 0.23. The minor allele frequency (MAF) ranged from 0.05 to 0.5, with an average of 0.15, and MAF > 0.5 was observed in 37.49% of the markers. Heterozygosity among the SNP markers varied from 0.04 to 0.62, with a mean of 0.11. The average polymorphic information content (PIC) was 0.20, with the highest and lowest values being 0.38 and 0.09, respectively (Table S4).

In addition, PCA and phylogenetic analysis were conducted using genic and non-genic markers distinctly. The PCA based on 35,347 genic SNPs explained 26.6% of the total genetic variation across the first two principal components (PC1 and PC2), while the PCA using 28,722 non-genic SNPs accounted for 28.7% of the total variance. In Figure S1, population 2 (red cluster) is tightly grouped, indicating high genetic similarity within that group, whereas population 1 (black cluster) shows a wider spread along both PC1 and PC2, which may reflect greater genetic diversity or gene flow. These results suggest that PCA based on overall markers (24,203 SNPs) was more informative for inferring genetic structure than genic or non-genic markers separately. Similarly, the dendrogram generated using 35,347 genic SNPs (Fig. S2A) and using 28,722 non-genic SNPs (Fig. S2B) was almost similar and captured all the major groupings as in the whole SNP data set. These results support the robustness of the genetic structure observed in our full SNP dataset and confirm that genic and non-genic SNPs alone also hold sufficient discriminatory power to reveal meaningful population structure.

### Application of AmahySNP in core collection development

A core set of amaranth germplasm was developed using SNP genotyping data from the AmahySNP array. Core Hunter 3 software was used to select core accessions at different sampling fractions to ensure representation of the genetic diversity present in the entire population. Two core sets, comprising 12% and 15% of the total accessions, were compared. The 15% core set showed reduced genetic diversity (GD) and minor allele frequency (MAF), whereas the 12% core set, consisting of 112 accessions, was considered more representative of the entire 917 accessions based on these parameters. The 12% core set included two selected varieties (Annapurna and Suvarna) and 100 accessions from 12 regions: 6 from the exotic collection, 9 from Gujarat, 44 from Himachal Pradesh, 3 from Madhya Pradesh, 6 from Maharashtra, 1 from Chhattisgarh, 2 from Tamil Nadu, 1 from Sikkim, 27 from Uttarakhand, 4 from Uttar Pradesh, and 9 with unknown origins (Table S5). The 24,203 SNP markers in the dataset generated ten types of alleles, four homozygous and six heterozygous (including 2 transitions and 4 transversions). The core set retained alleles with nearly identical frequencies (99.9% similarity) and showed no allele loss compared to the entire collection (Table S6, Fig. S3).

### Genetic diversity assessment of core collection

**Fig. 5:**
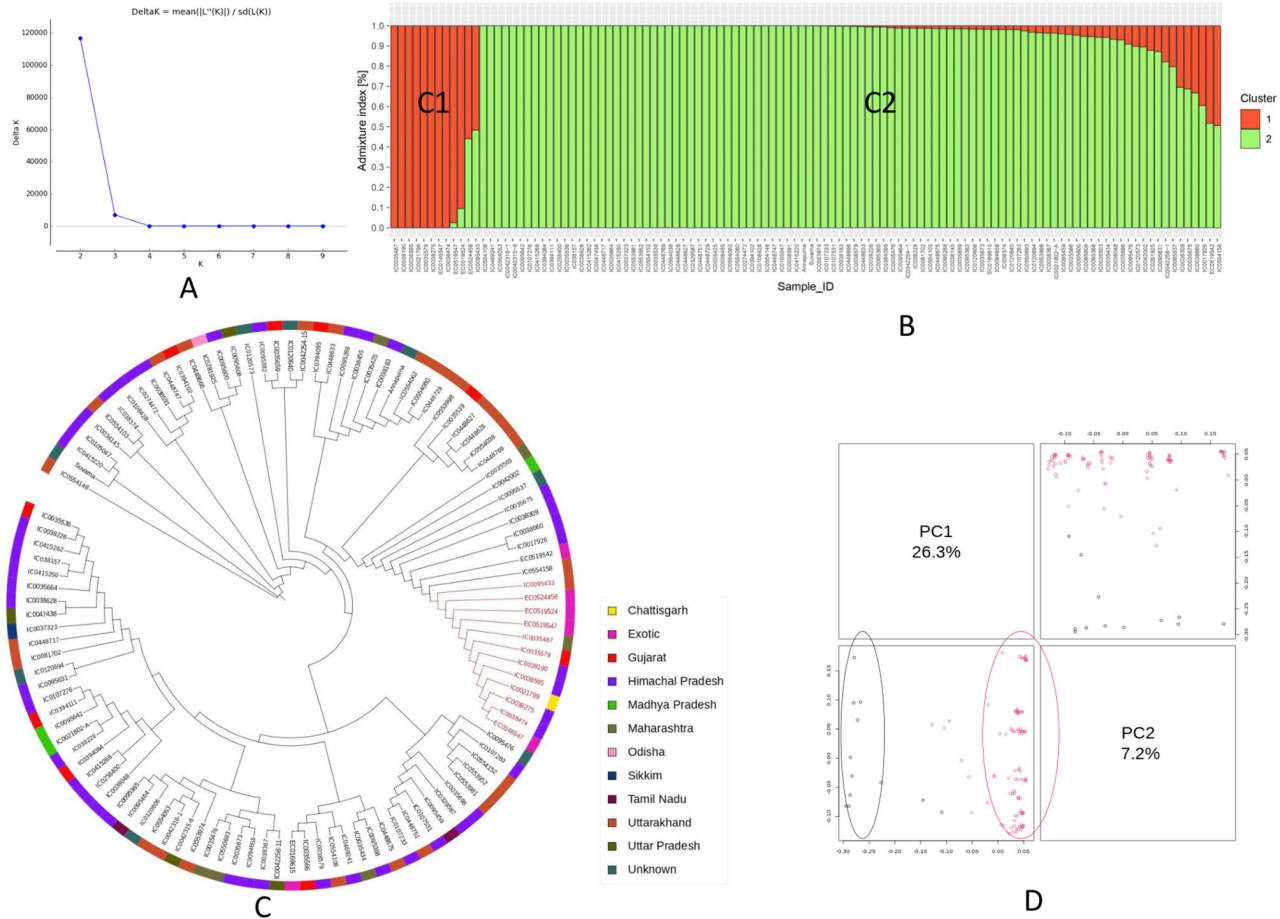
Genetic diversity analysis of core set (**A**): Plot of a subpopulation (K) against delta K (**B**): Structure plot for 112 genotypes at K = 2 such that each colour represents one subpopulation, namely C1 and C2 (**C**): Neighbour-joining phylogenetic tree of 112 core set accessions based on genotyping of the AmahySNP assay. The outer color strip represents the different geographical locations of each accession, and the black and red branches represent C1 and C2 subpopulations, respectively (**D**): Principal component analysis on 112 genotypes grouped into two clusters corresponding to C1 and C2

Kinship analysis revealed low genetic relatedness within the amaranth core collection, indicating that it is an ideal core set for molecular and genetic studies (Fig. S4). To assess the reliability of the core set, its diversity parameters, including GD, Ho, PIC, and MAF, were compared with those of the entire collection. The results showed similar values between the core set and the entire collection, confirming that the selected 112 accessions accurately represented the whole population (Table S4). STRUCTURE analysis clustered the core set into two populations, as the maximum likelihood was estimated to be greater at (k = 2) (Fig. 5A). Population 1 and Population 2, designated as C1 and C2, respectively, comprised 10 pure and 2 admixed accessions in C1, and 94 pure and 6 admixed accessions in C2 (Fig. 5A and B). Phylogenetic analysis further divided the core set into three clusters: Cluster I (1 genotype), Cluster II (3 genotypes), and Cluster III (108 genotypes) (Fig. 5C). PCA analysis revealed that PC1 and PC2 accounted for 26.3% and 7.2% of the total variance, respectively, which was consistent with the subpopulations identified in the STRUCTURE analysis (Fig. 5D). The diversity pattern of the core set closely mirrored that of the entire population, confirmed its reliability as a representative subset of the 917 accessions.

### Utility of the AmahySNP assay in GWAS analysis for DTF

To assess the effectiveness of the AmahySNP array for genome-wide association analysis, we analyzed 64 K-SNP genotyping data from 540 accessions selected from the initial pool of 917 accessions, which were phenotyped for the DTF trait during the Kharif (E1) and Rabi (E2) seasons (Table S7). Significant phenotypic variation was observed, with DTF ranging from 26 to 64 days with an average of 38 days in E1 and 30–64 days with an average of 34 days in E2 (Table S8). Due to significant heterogeneity in DTF error variances, independent analyses were conducted for each environment. Correlations between the two environments were evaluated to understand the genotypic response for trait expression. A statistically significant positive correlation was observed with a Pearson correlation coefficient (PCC = 0.25, *p* < 0.001) between E1 and E2, indicating a consistent genotypic response for the DTF trait across environments (Fig. S5).

**Fig. 6:**
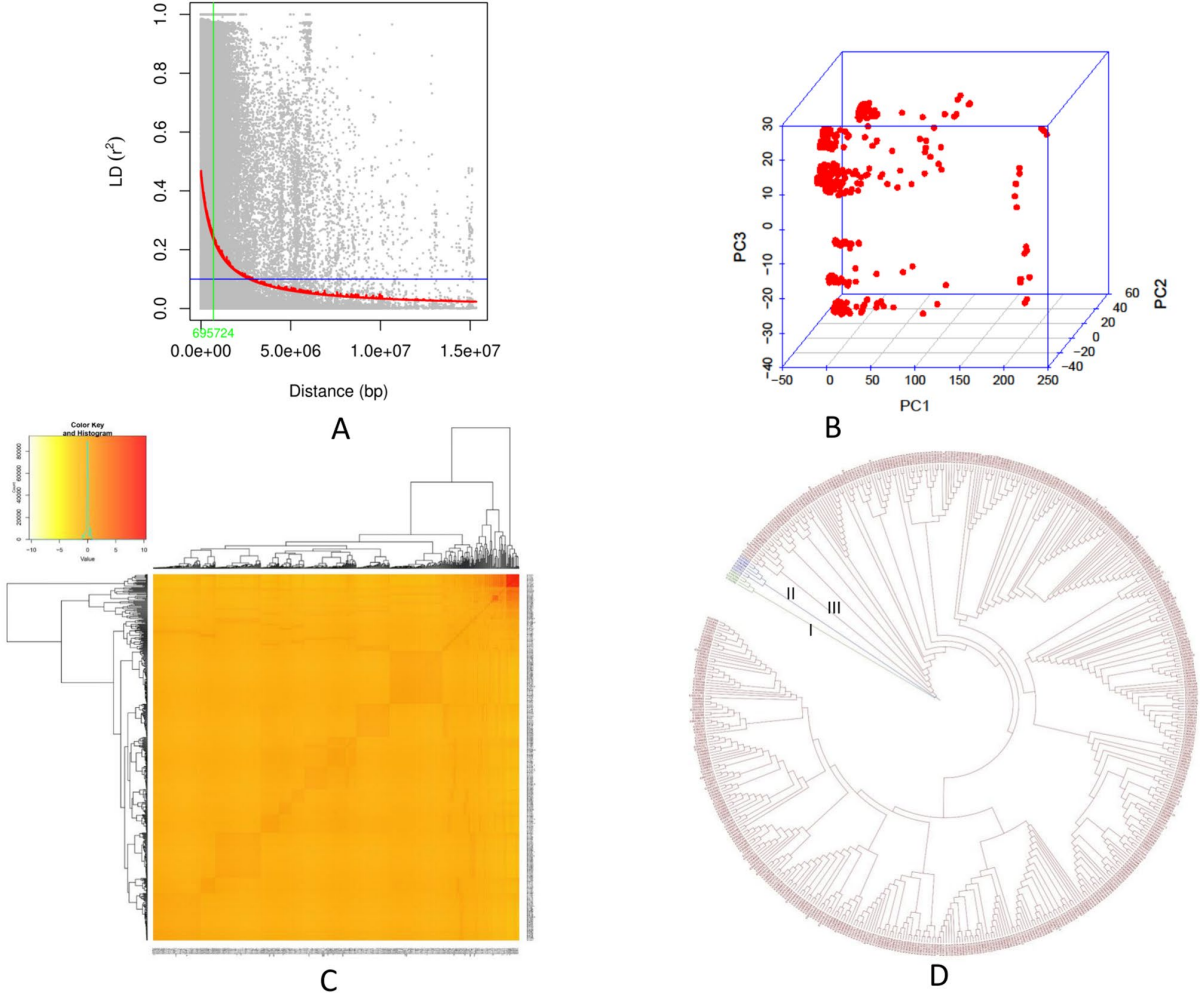
Linkage disequilibrium decay pattern and genetic structure of the 540 amaranth genotypes. (**A**) LD decay plot based on 540 accessions and 16 scaffolds of the amaranth genome to determine the linkage disequilibrium distance; the red line indicates the LD decay trend. (**B**) Principal component analysis depicting clustering of the 540 genotypes. (**C**) Pairwise kinship Heat map illustrating the relatedness of 540 amaranth accessions with the dendrogram shown on top and left; the figure in the infix represents the color coding of the structured heat map and frequency curve of kinship values among the selected genotypes. (**D**) A phylogenetic tree of 540 amaranth genotypes was constructed using 22,763 polymorphic SNPs from the 64k SNP chip, grouped into three clusters, namely Cluster I, II, and III

The genome-wide LD decay in the amaranth genome followed a nonlinear trend with respect to physical distance, with an LD decay distance of 695.72 kb where *r*^2^ decreased to half its maximum value (Fig. 6A). The average LD across scaffolds was *r*^2^ = 0.68; 2.32% of SNP pairs were in complete LD (*r*^2^ = 1), and 74.2% exhibited strong LD (*r*^2^ value > 0.5). Kinship analysis revealed low genetic relatedness among the 540 accessions, suggesting that the panel is suitable for GWAS. LD decay was further assessed using 22,763 SNPs, with the squared correlation coefficient (*r*^2^) calculated in TASSEL software (Fig. 6C). Phylogenetic analysis of 540 accessions using the neighbor-joining algorithm identified three clusters: Cluster I (4 accessions, 0.74%), including three indigenous and one exotic genotype; Cluster II (6 accessions, 1.11%), all indigenous; and Cluster III (530 accessions, 98.15%), which was the largest cluster (Fig. 6D).

Two SL-GWAS methods (MLM and CMLM) and four ML-GWAS methods (mrMLM, FASTmrMLM, FASTmrEMMA, and pLARmEB) were used to identify genomic regions/QTNs associated with the DTF trait in environments E1 and E2. Stable SNPs were defined as those detected by at least two methods simultaneously. In E1, SL-GWAS identified 34 significant SNPs, while ML-GWAS identified 31. For E2, SL-GWAS identified 76 significant SNPs, and ML-GWAS identified 30. Across both environments, 22 significant QTNs with (LOD ≥ 3) were identified using at least two ML-GWAS methods. In E1, 11 QTNs associated with DTF (qDTF-1-1, qDTF-1-2, qDTF-1-3, qDTF-4-1, qDTF-4-2, qDTF-5-1, qDTF-7-1, qDTF-7-2, qDTF-8-1, qDTF-14-1, and qDTF-16-1) were located on scaffolds 1, 4, 5, 7, 8, 14, and 16. In E2, 11 QTNs (qDTF-1-1, qDTF-1-2, qDTF-1-3, qDTF-2-1, qDTF-6-1, qDTF-7-1, qDTF-7-2, qDTF-9-1, qDTF-10-1, qDTF-13-1, and qDTF-13-2) were distributed on scaffolds 1, 2, 6, 7, 9, 10, and 13. Thirteen QTNs were common between SL-GWAS and ML-GWAS (LOD ≥ 3), with one QTN on scaffold 7 at 16,342,396 bp associated with DTF in both environments (Table S8). A total of seven and six QTNs were associated with DTF in E1 and E2, respectively. These identified QTNs were subjected to further analyses.

**Fig. 7:**
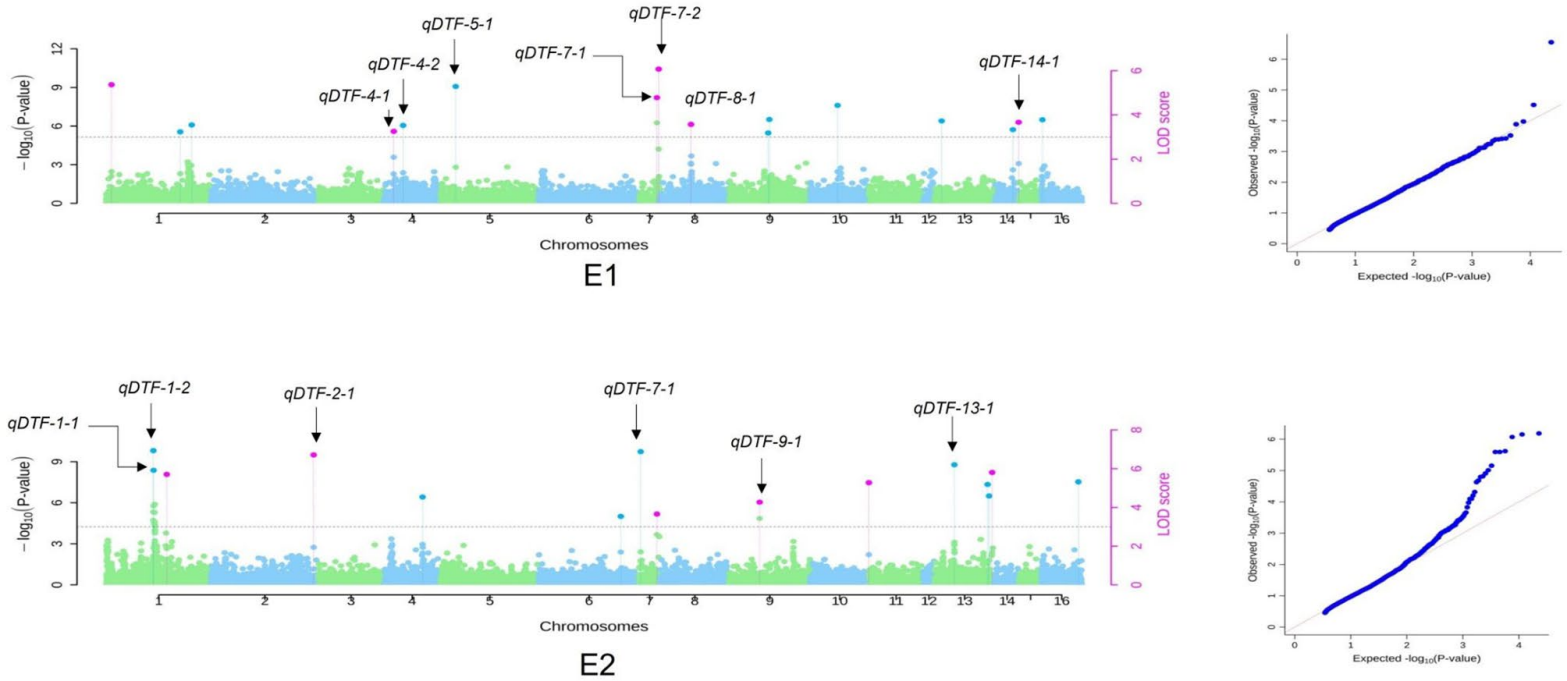
GWAS for DTF trait in amaranth accessions. Manhattan and Quantile-Quantile plots derived through mrMLM, FASTmrMLM, FASTmrEMMA, and pLARmEB methods depict significant marker-trait associations on 16 scaffolds of amaranth for the years (**A**) 2019–2020 and (**B**) 2020–2021. QTNs commonly identified by various GWAS methods with LOD ≥ 3 are indicated by the pink dots above the dotted vertical lines, while all the QTNs identified by one single method are indicated by the blue dots

Manhattan and quantile-quantile (QQ) plots are shown in Fig. 7, depicting QTNs with LOD ≥ 3 identified through single- and multi-locus models in environments E1 and E2 (Fig. 7A and B). In both environments, the observed *P*-values corresponded closely to the expected *P*-values, as all the points were located on or near the middle line in the QQ plots. However, in E2, the observed *P*-values were more significant than expected, in comparison to E1.

### Annotation of identified stable QTNs

All 13 novel genomic loci associated with DTF were annotated using the *A. hypochondriacus* genome assembly v2.1 from Phytozome 13. Of these, 7 QTNs were found near annotated genes, while the remaining were intergenic SNPs. Among the annotated QTNs, one QTN located on chromosome 7 at position 16,342,396 bp was identified by five different methods across both E1 and E2 environments. This QTN encodes for a zinc knuckle family protein (LOC_Os12g37720). In E1, qDTF-4-2 (AX-587783867) was linked to a gene encoding a zinc finger RING-type protein, and a transcription factor homologous to AT4G21430, which promotes flowering through interaction with FBH transcription factors [56]. Another QTN, qDTF-5-1 (AX-587790101), located on scaffold 5 at position 2,338,055 bp, was annotated as an ABA/WDS-induced protein (AH008526), homologous to LOC_Os06g12580. This protein regulates floral organ development by interacting with key proteins, including OsPID, LAX1, and OsMADS16 [57]. In E2, qDTF-1-1, qDTF-9-1, and qDTF-13-1 were associated with genes encoding an expressed protein, ribosomal protein 60 S L5/18, and an oxidation resistance protein, respectively. QTN qDTF-1-1, located on scaffold 1 at position 22,191,367 bp, was homologous to LOC_Os04g40700, which encodes a steroid nuclear receptor. QTN qDTF-9-1 (AX-587828210), located on scaffold 9 at position 12,602,621 bp, was identified by five different methods: MLM, CMLM, mrMLM, pLARmEB, and FASTmrMLM within the gene AH014416, homologous to Cre14.g621450, and annotated as RIBOSOMAL PROTEIN L5-RELATED. QTN qDTF-13-1 (AX-587862355) was linked to gene AH019886, a TLD-domain-containing nucleolar protein homologous to AT2G05590, which is involved in oxidative stress responses.

### Candidate gene identification of stable QTNs

Genes within a 695 kb region upstream and downstream of six stable and unique QTNs were analyzed to identify potential candidate genes based on functional annotations and homology with Arabidopsis and Oryza orthologs. A total of 237 genes were identified near the stable QTNs, of which 54 were associated with the DTF trait (Table S9). Key putative genes related to flowering time traits include *AH019869* (FAR1 DNA-binding domain), *AH019896* (PLATZ transcription factor), *AH019873* (GATA transcription factor 15-related), *AH019875* (protein KINESIN light chain-related), *AH014379* (Zinc finger protein CONSTANS-like), *AH014397* (Auxin-responsive protein IAA), *AH004592* (F-box/kelch-repeat protein SKIP11), *AH008516* (Tubby-like F-box protein 1-related), *AH008547* (GIGANTEA), *AH011804* (ubiquitin-protein ligase E3 C), and *AH011815* (AGAMOUS-like MADS-box protein AGL36-related).

## Discussion

Molecular markers are essential tools in genomics-assisted breeding (GAB), facilitating the characterization and utilization of crop genetic resources for efficient varietal improvement [58]. Various marker systems, including RFLPs, SSRs, RAPDs, ISSRs, AFLPs, and SNPs, have been utilized in genetic research and breeding, with SNPs being the most abundant and uniformly distributed, providing highly effective and precise genetic analysis [59, 60]. High-density SNP genotyping assays facilitate rapid profiling of numerous markers, aiding the dissection of complex traits and accelerating crop improvement [61, 62]. Compared to fixed high-density SNP arrays, the GBS approach is less effective as it does not generate allelic data for all detected SNPs in a given population, requires high-performance computing and advanced programming skills, and has lower reproducibility than SNP arrays and whole-genome sequencing [30, 63]. Recently, the development of over 50 SNP-based arrays and 15 GBS platforms across more than 25 crop species exemplifies the growing technological momentum in this domain. While GBS is cost-effective and facilitates SNP discovery, it is limited by incomplete allelic data generation, computational demands, and lower reproducibility compared to SNP arrays and whole-genome sequencing. SNP arrays, by contrast, deliver high-throughput, predictive, and reproducible genotyping with broad applications in GWAS and molecular breeding [64]. In crops like kiwifruit, the newly developed 135 K SNP array enabled high-resolution genetic mapping, QTL analyses, and effective performance in polyploid populations [65]. Similarly, the 580 K SNP chip in rice has facilitated fine-scale trait mapping and prediction models for agronomic improvements [66]. These advances underscore the transformative impact of high-density SNP arrays across diverse crop systems. To date, only a few studies on amaranth have been published, including SNP identification through genomic reduction [35], two-enzyme digestion [67], and the GBS approach [37]. To address this gap in amaranth germplasm characterization, we developed the AmahySNP 64 K array for *A. hypochondriacus*, comprising 64,069 SNPs including 35,347 genic SNPs from 8,879 genes and 28,722 intergenic SNPs, achieving QC call rate of ≥ 99.3% and an average marker density of ~ 1 SNP per 6.17 kb, sufficient for population structure analysis, genetic diversity assessment, and GWAS. A transition-to-transversion ratio of 1.52 indicated a bias toward transition mutations (A/G and C/T), potentially linked to methylation, which is consistent with other crops like wheat, barley, and tomato [68–70]. Transition SNP mutations have been favoured in SNP array development [71, 72]. The 64 K SNP array, AmahySNP, demonstrated diverse applications, including population structure and genetic diversity analyses, core set development, and GWAS for DTF traits, underscoring its potential to transform agricultural research and crop breeding.

The ICAR-NBPGR, in India, maintains the largest germplasm collection of amaranths (5,347 grain and 571 vegetable accessions from 41 species), yet its utility in breeding programs has been hampered due to a lack of molecular characterization [73]. Therefore, genotypic and agronomic profiling of these accessions is essential for developing new varieties and hybrids. The AmahySNP array provides a robust tool for genotyping diverse *A. hypochondriacus* accessions. By using the AmahySNP array, we genotyped 917 amaranth accessions to analyze population structure, genetic diversity, and core collection development. The SNPs data generated with the 64 K array were filtered based on MAF ≥ 0.05, and observed higher SNP densities in pericentromeric regions than in distal ends. Population structure analysis revealed two subpopulations with evidence of admixture in some accessions, essential for avoiding false GWAS associations via population stratification correlation [74]. The parameters like gene diversity (GD), heterozygosity, and PIC provided insights into the evolutionary dynamics of alleles within a given breeding population and the mutation rate at a specific locus over time [75]. Genetic diversity assessment revealed an average gene diversity (GD) of 0.23, exceeding the average PIC value (0.20), indicating a moderate level of allelic diversity at the SNP loci. The PIC ranged from 0.09 to 0.38 with an average of 0.20, indicating moderate to low informativeness due to the biallelic nature of the SNP markers [76]. Despite being moderately informative, the observed PIC values were relatively higher than those observed in earlier amaranth studies [77], while the low observed heterozygosity (0.11) reflects the self-pollinating nature of the crop [78]. It suggests that the accessions are predominantly inbred or genetically fixed; amaranth is a self-pollinating species subjected to several generations of purifying selection bottlenecks. It also indicates limited recent outcrossing among the accessions. We established a core collection representing ~ 12% of the accessions that maintained high genetic diversity, low heterozygosity, and low redundancy, valuable for breeding and germplasm characterization [79]. Core collections for various crops, including *Amaranthus tricolor*, *Brassica napus*, *Cucumis sativus*, and *Glycine max*, have been designed [80–83]. The amaranth core collection captured the genetic diversity of the total population, offering a valuable resource for breeders to improve this important crop.

The AmahySNP array was used for GWAS on days to 50% flowering (DTF) using data from two contrasting seasons: Rabi (winter) and Kharif (summer) seasons in New Delhi. In DTF analysis, the coefficient of variation was 19.84 for E1 and 16.78 for E2, indicating high genotypic variation. The results showed that all genotypes in both E1 and E2 environments flowered early (< 70 days), with no late flowering accessions (> 80 days), likely due to the presence of photoperiod-insensitive accessions from higher altitudes [84]. Additionally, early flowering in E1 and E2 was influenced by drought and high temperatures, which halts the leaf production. Early flowering in amaranth is considered a desirable trait in high-altitude regions. To demonstrate the utility of the AmahySNP chip, a GWAS for the DTF traits was performed using two single-locus and four multi-locus models. Multi-locus models overcome the limitations of single-locus GWAS by reducing false positives. Only a few genes controlling DTF in grain amaranth have been reported, with one previous study reported six potential candidate genes associated with flowering time [78]. In this study, the aanalyses of 540 amaranth accessions identified six stable QTNs, with 54 candidate genes detected near these QTNs in E1 and E2. Key genes associated with DTF included *AH007573* (Jumonji domain-containing protein), carrying QTN qDTF-4-2 within the gene sequence and regulating flowering via the CONSTANS (CO) transcription factor and its interaction with FLOWERING BHLH (FBHs). AH001195 (expressed protein) carrying QTN qDTF-1-1, a homolog of *AT3G27390*, showed upregulation in flowering tissue, suggesting its role in flowering time [85]. AH007579 (Tudor/PWWP/MBT superfamily protein), an ortholog of *AT3G05430.1*, interacts with POLYCOMBS1 (PWO1) and PRC2, lowering FLOWERING LOCUS C (FLC) expression to promote early flowering [86]. Other significant genes included *AH019919* (COP1-interacting protein), which represses photoperiodic flowering through CO degradation via ubiquitin ligase activity, and *AH014379* (ZINC FINGER CONSTANS-LIKE 5), which regulates FLOWERING LOCUS T expression [87, 88]. Multiple F-box proteins, such as AH007559, AH007572, and AH008516, were identified, known for their role in floral organ determination, self-incompatibility, floral meristem identity, and circadian clock regulation [89, 90]. Additionally, AH019875 (KINESIN LIGHT CHAIN-RELATED 3-like) was linked to pollen development, while AH011815 (AGAMOUS-LIKE MADS-BOX PROTEIN) and other transcription factors (AH019874, AH019896, AH019900) emerged as key regulators of flowering. Overall, this study demonstrates the effectiveness of the AmahySNP array in genetic diversity analysis, population structure determination, core collection creation, and GWAS, proving its utility in advancing amaranth breeding and genetics.

## Conclusions

The genus *Amaranthus* comprises several species cultivated as versatile, multi-purpose crops. In this study, we developed a 64 K high-density SNP array named “AmahySNP” using SNPs identified through whole-genome resequencing of *A. hypochondriacus* genotypes originating from various countries. This array was utilized for core set development, genetic diversity assessment, population structure analysis, and GWAS in a diverse amaranth panel. GWAS analysis for the DTF trait demonstrated that the chip can be effectively used in identifying potential candidate genes to enhance molecular-assisted breeding in *A. hypocondriacus*. With a high call rate of 99.3%, the array showed excellent reproducibility, accuracy, and cost-effectiveness due to its optimized SNP density and distribution throughout the genome. Overall, the AmahySNP array has significant potential for advancing the genetic and molecular understanding of *A. hypocondriacus*, thereby providing valuable insights for crop improvement programs and enabling better utilization of this underutilized crop.

## Supplementary Information

Supplementary Material 1. Figure S1: Principal component analysis (PCA) using genic (35,347) and non-genic (28,722) SNPs. Figure S2: Phylogenetic tree generated using genic (35,347) and non-genic (28,722) SNPs. Figure S3: Graph showing the allele frequency of 917 genotypes in the amaranth panel and core collection. Figure S4: Pairwise kinship heat map illustrating the relatedness within the core collection (112 amaranth accessions) with the dendrogram shown on top and left; the figure in the infix represents the color coding of the structured heat map and frequency curve of kinship values among the selected genotypes. Figure S5: Distribution and Pearson correlation coefficient analysis. of the DTF trait in two environments, E1 and E2.

Supplementary Material 2. Table S1: Details of 917 *A. hypochondriacus* accessions used in the present study. Table S2: Distribution of SNP loci in the AmahySNP array in the amaranth genome. Table S3: Single-nucleotide polymorphism statistics and frequency of allele occurrence in the 64k SNP chip, where (A: G) indicates that A is a reference allele and G is the alternate allele. Table S4: Comparative list of genetic diversity indices estimated for the total collection and core sets. Table S5: List of 112 Amaranth core set accessions. Table S6: List of allele frequencies of total collection and core collection. Table S7: List of 540 grain amaranth accessions used for the GWAS study. Table S8: List of significant QTNs for DTF traits detected simultaneously using SL-GWAS and ML-GWAS methods in two environments, E1 and E2. Table S9: List of 54 potential candidate genes identified as associated with the DTF trait.

## Data Availability

The datasets supporting the conclusions of this article are included within the article and its supplementary files.
