# Correction: Development and validation of a highdensity ‘Amahysnp’ genotyping array in grain Amaranth (Amaranthus hypochondriacus)

**DOI:** 10.1186/s12870-025-07853-4

**Published:** 2025-12-06

**Authors:** Rakesh Singh, Ajay Kumar Mahato, S. Rajkumar, A.K. Singh, Akshay Singh, Avantika Maurya, Rajat Gupta, Rakesh Bhardwaj, S. K. Kaushik, Sandeep Kumar, Veena Gupta, Kuldeep Singh, G. P. Singh

**Affiliations:** 1 Division of Genomic Resources, ICAR-National Bureau of Plant Genetic Resources, Pusa Campus, New Delhi, India; 2 Centre for DNA Fingerprinting and Diagnostics, Hyderabad, India; 3 Division of Germplasm Evaluation, ICAR- National Bureau of Plant Genetic Resources, Pusa Campus, New Delhi, India; 4 Division of Germplasm Conservation, ICAR- National Bureau of Plant Genetic Resources, Pusa Campus, New Delhi, India; 5 International Crops Research Institute for the Semi-Arid Tropics, Patancheru, Telangana India; 6 ICAR-National Bureau of Plant Genetic Resources, Pusa Campus, New Delhi, India

**Correction: BMC Plant Biol 25**,** 1281 (2025)**

**https://doi.org/10.1186/s12870-025-07367-z**

Following publication of the original article [1], the authors found errors in the capturing of the two author names. The details are as follows:

- Current author names read as: A. Singh, Akshay K. Singh
- Correct author names should be: **A.K. Singh**,** Akshay Singh**

The corrections do not affect the conclusion of the article. The original article [1] has been corrected.
