# Supplementary material for: Development and validation of a high-density ‘Amahysnp’ genotyping array in grain amaranth (Amaranthus hypochondriacus)
Source: BMC Plant Biol. 2025 Oct 1;25:1281. doi: 10.1186/s12870-025-07367-z (PMC12487340; doi:10.1186/s12870-025-07367-z)
Supplement: Supplementary file 1 — Supplementary Material 1. Figure S1: Principal component analysis (PCA) using genic (35,347) and non-genic (28,722) SNPs. Figure S2: Phylogenetic tree generated using genic (35,347) and non-genic (28,722) SNPs. Figure S3: Graph showing the allele frequency of 917 genotypes in the amaranth panel and core collection. Figure S4: Pairwise kinship heat map illustrating the relatedness within the core collection (112 amaranth accessions) with the dendrogram shown on top and left; the figure in the infix represents the color coding of the structured heat map and frequency curve of kinship values among the selected genotypes. Figure S5: Distribution and Pearson correlation coefficient analysis. of the DTF trait in two environments, E1 and E2. [file 12870_2025_7367_MOESM1_ESM.docx]

**Supplementary Figures**

**
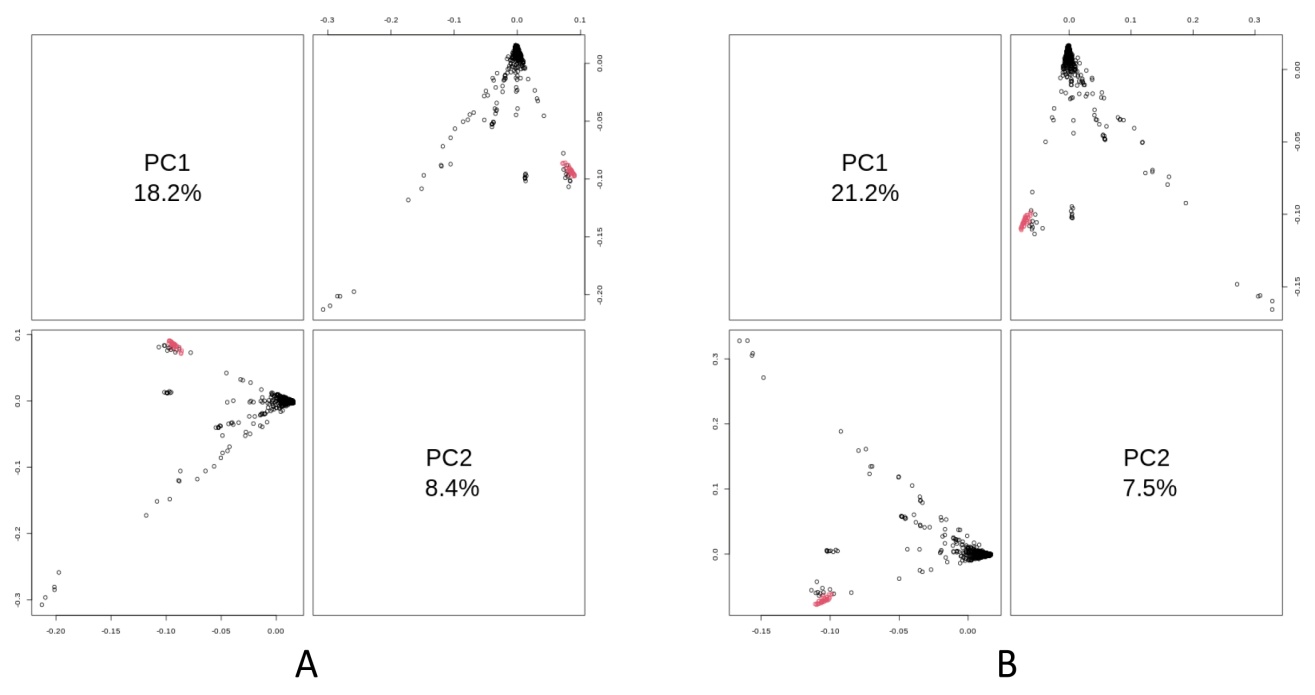
Fig. S1.** Principal component analysis (PCA) plots generated (A) using 35,347 genic SNPs and (B) using 28,722 non-genic SNPs.


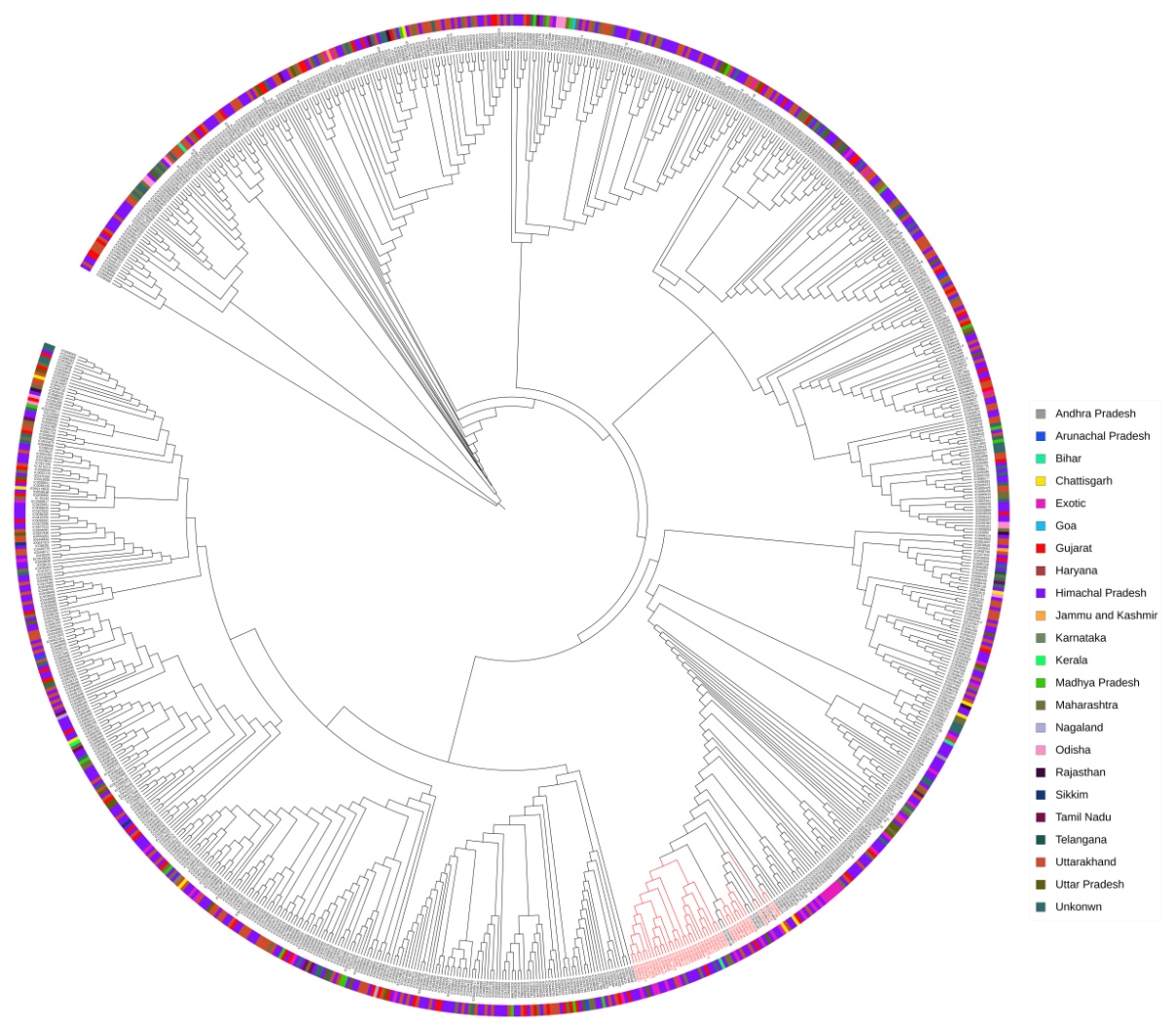


(A)


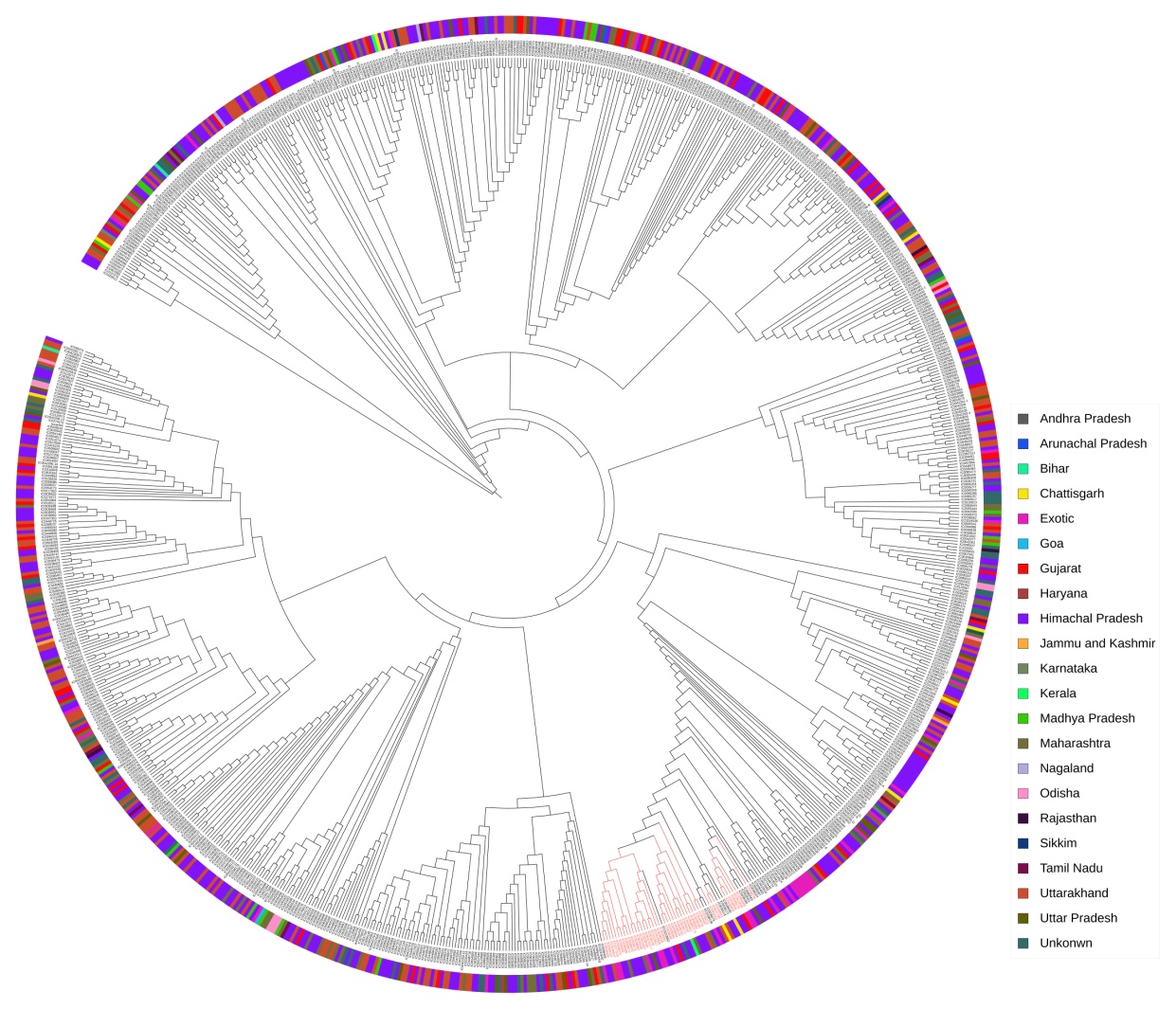


(B)

**Fig. S2.** Phylogenetic tree generated (A) using 35,347 genic SNPs and (B) using 28,722 non-genic SNPs. The outer color strip illustrates the different geographical locations of each accession, and the black and red branches represent the accessions from the P1 and P2 subpopulations, respectively


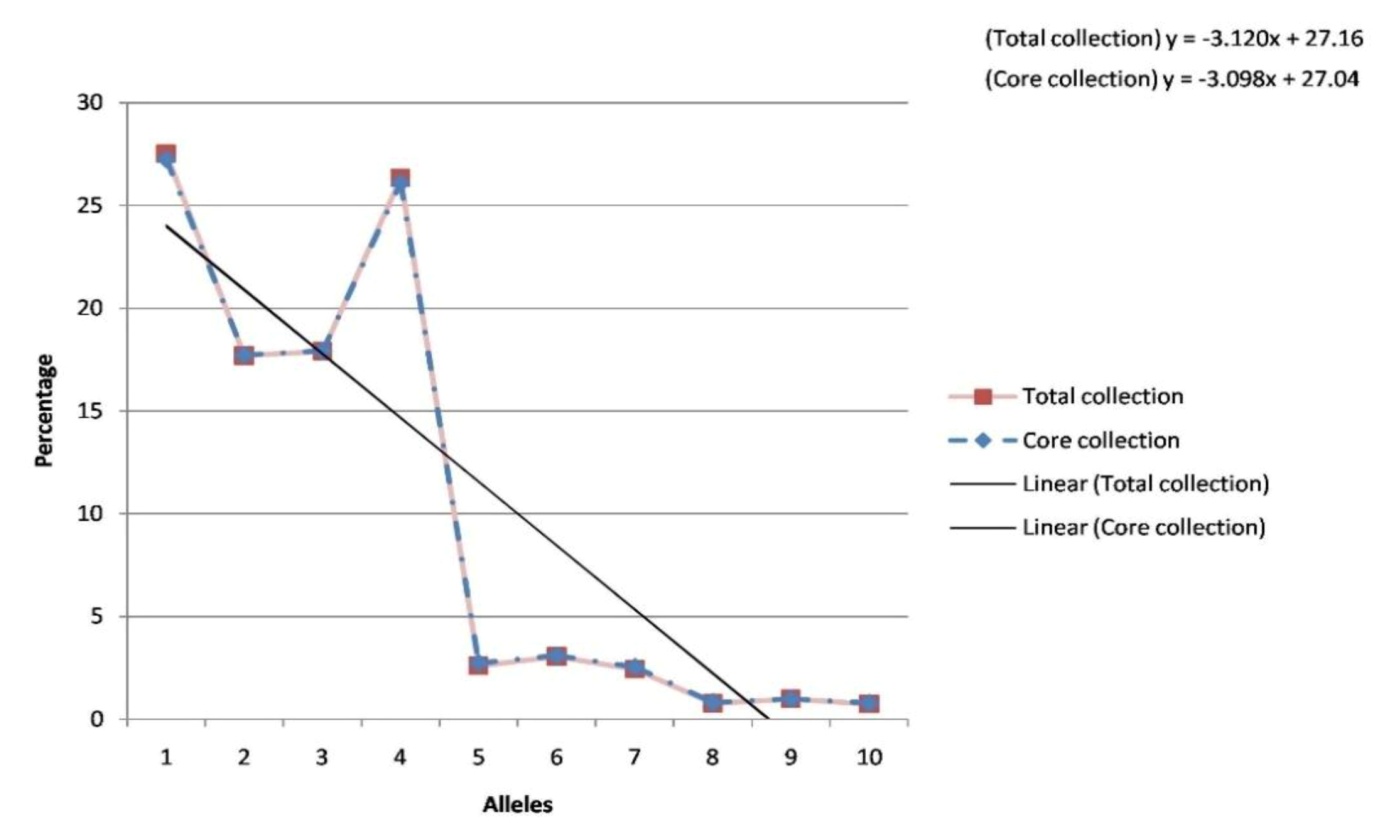


**Fig. S3:** Graph showing the allele frequency of 917 genotypes in the amaranth panel and core collection.


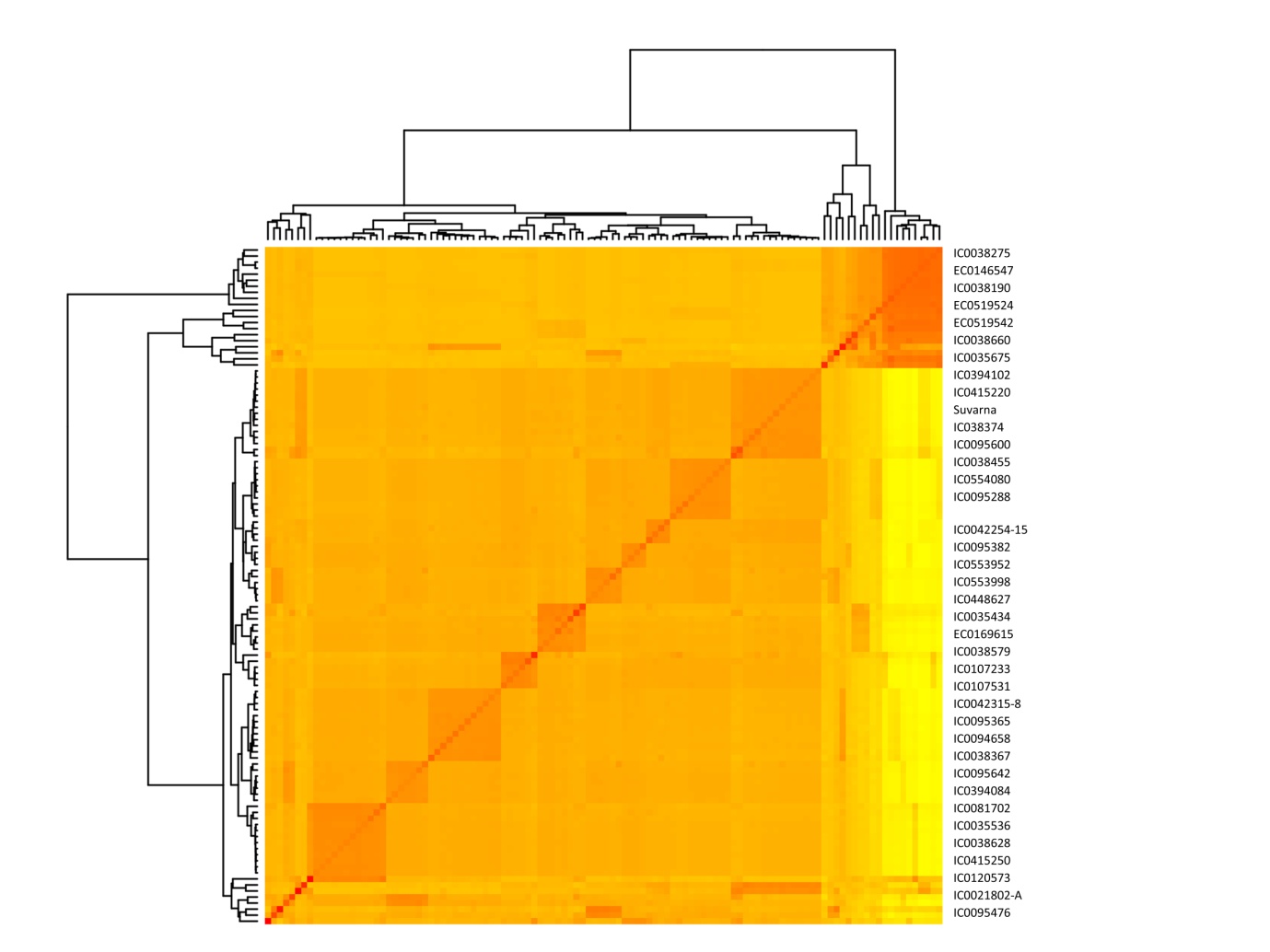


**Fig. S4:** Pairwise kinship heat map illustrating the relatedness within the core collection (112 amaranth accessions) with the dendrogram shown on top and left; the figure in the infix represents the color coding of the structured heat map and frequency curve of kinship values among the selected genotypes.


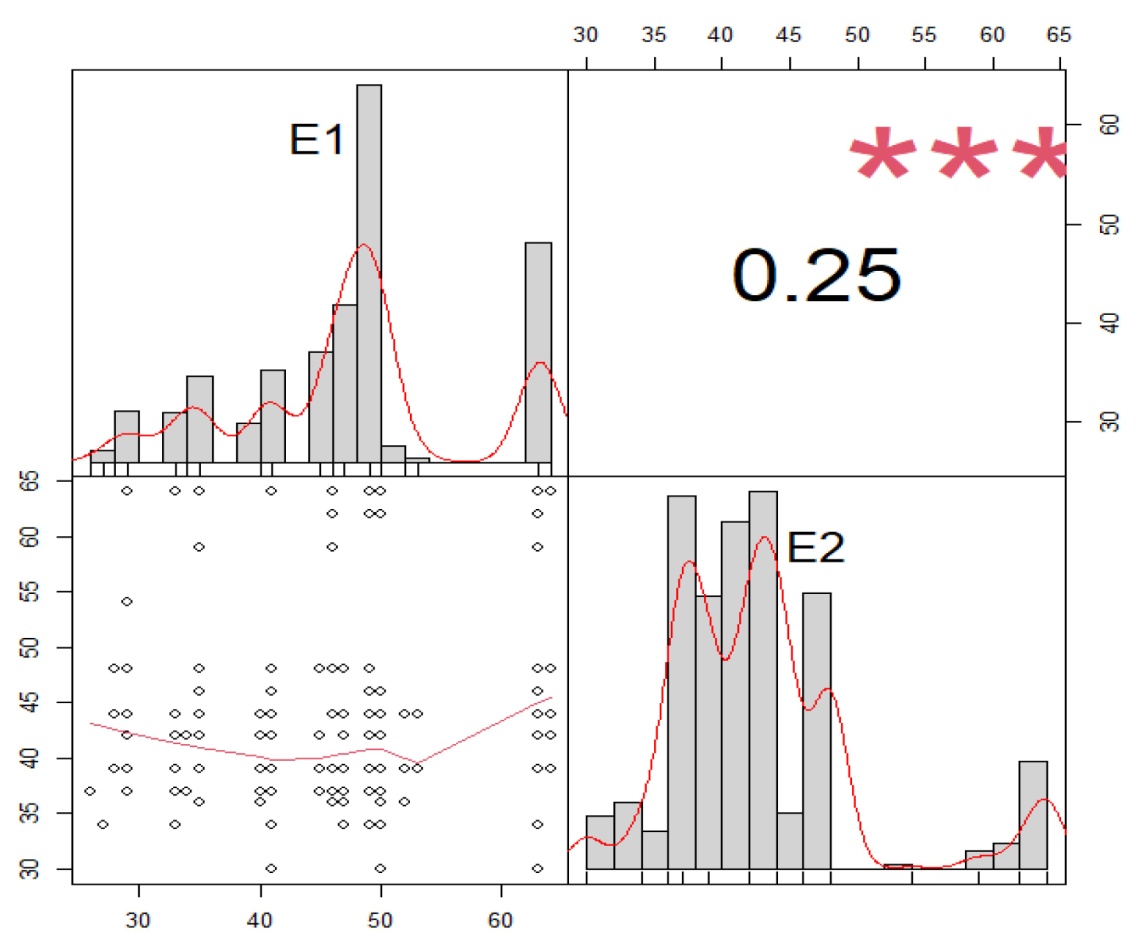


**Fig. S5:** Distribution and Pearson correlation coefficient analysis. of the DTF trait in two environments, E1 and E2.
