# Supplementary material for: Development and validation of a high-density ‘Amahysnp’ genotyping array in grain amaranth (Amaranthus hypochondriacus)
Source: BMC Plant Biol. 2025 Oct 1;25:1281. doi: 10.1186/s12870-025-07367-z (PMC12487340; doi:10.1186/s12870-025-07367-z)
Supplement: Supplementary file 2 — Supplementary Material 2. Table S1: Details of 917 A. hypochondriacus accessions used in the present study. Table S2: Distribution of SNP loci in the AmahySNP array in the amaranth genome. Table S3: Single-nucleotide polymorphism statistics and frequency of allele occurrence in the 64k SNP chip, where (A: G) indicates that A is a reference allele and G is the alternate allele. Table S4: Comparative list of genetic diversity indices estimated for the total collection and core sets. Table S5: List of 112 Amaranth core set accessions. Table S6: List of allele frequencies of total collection and core collection. Table S7: List of 540 grain amaranth accessions used for the GWAS study. Table S8: List of significant QTNs for DTF traits detected simultaneously using SL-GWAS and ML-GWAS methods in two environments, E1 and E2. Table S9: List of 54 potential candidate genes identified as associated with the DTF trait. [file 12870_2025_7367_MOESM2_ESM.docx]

**Supplementary Table 1.** Details of 917 *A. hypochondriacus* accessions used in present study.

| **S. No.** | **Accession No.** | **State** | **Cultivar Name** | **Year** |
| --- | --- | --- | --- | --- |
| 1 | IC0095624 | bihar |  | - |
| 2 | IC0038040 | chattisgarh | bilaspur-1 | 1980 |
| 3 | IC0021926 | chattisgarh |  | 1974 |
| 4 | IC0618487 | chattisgarh |  | 2016 |
| 5 | IC0021796-B | chattisgarh |  | 1974 |
| 6 | IC0021927 | chattisgarh |  | 1974 |
| 7 | IC0021966 | chattisgarh |  | 1974 |
| 8 | IC0026265 | goa |  | 1976 |
| 9 | IC0398215 | gujarat |  | 2003 |
| 10 | IC0398217 | gujarat |  | 2003 |
| 11 | IC0398237 | gujarat |  | 2003 |
| 12 | IC0394084 | gujarat |  | 2003 |
| 13 | IC0394087 | gujarat |  | 2003 |
| 14 | IC0394091 | gujarat |  | 2003 |
| 15 | IC0394092 | gujarat |  | 2003 |
| 16 | IC0394095 | gujarat |  | 2003 |
| 17 | IC0394096 | gujarat |  | 2003 |
| 18 | IC0394097 | gujarat |  | 2003 |
| 19 | IC0394102 | gujarat |  | 2003 |
| 20 | IC0394108 | gujarat |  | 2003 |
| 21 | IC0035511 | gujarat |  | 1979 |
| 22 | IC0035518 | gujarat |  | 1979 |
| 23 | IC0035519 | gujarat |  | 1979 |
| 24 | IC0035528 | gujarat |  | 1979 |
| 25 | IC0035530 | gujarat |  | 1979 |
| 26 | IC0035534 | gujarat |  | 1979 |
| 27 | IC0035536 | gujarat |  | 1979 |
| 28 | IC0035539 | gujarat |  | 1979 |
| 29 | IC0035541 | gujarat |  | 1979 |
| 30 | IC0035543 | gujarat |  | 1979 |
| 31 | IC0035548 | gujarat |  | 1979 |
| 32 | IC0035551 | gujarat |  | 1979 |
| 33 | IC0035552 | gujarat |  | 1979 |
| 34 | IC0035553 | gujarat |  | 1979 |
| 35 | IC0035557 | gujarat |  | 1979 |
| 36 | IC0035558 | gujarat |  | 1979 |
| 37 | IC0035774 | gujarat |  | 1979 |
| 38 | IC0038534 | gujarat |  | 1979 |
| 39 | IC0095277 | himachal pradesh |  | 1962 |
| 40 | IC0278912 | himachal pradesh |  | 2000 |
| 41 | IC0095394 | himachal pradesh |  | 2001 |
| 42 | IC0038045 | himachal pradesh | arki-14 | 1980 |
| 43 | IC0038044 | himachal pradesh | arki-4 | 1980 |
| 44 | IC0038458 | himachal pradesh | bakrote-2 | 1980 |
| 45 | IC0038464 | himachal pradesh | bakrote-8 | 1980 |
| 46 | IC0038297 | himachal pradesh | Banda-2 | 1980 |
| 47 | IC0038299 | himachal pradesh | Banda-4 | 1980 |
| 48 | IC038303 | himachal pradesh | Banda-8 | 1980 |
| 49 | IC0038405 | himachal pradesh | banera-1 | 1980 |
| 50 | IC0038406 | himachal pradesh | banera-2 | 1980 |
| 51 | IC0038408 | himachal pradesh | banera-4 | 1980 |
| 52 | IC0038614 | himachal pradesh | barelengi-3 | 1980 |
| 53 | IC0038123 | himachal pradesh | Bawri-4 | 1980 |
| 54 | IC0038124 | himachal pradesh | bawri-5 | 1980 |
| 55 | IC0038647 | himachal pradesh | bekhalti-2 | 1980 |
| 56 | IC0038648 | himachal pradesh | Bekhalti-3 | 1980 |
| 57 | IC0038650 | himachal pradesh | bekhalti-5 | 1980 |
| 58 | IC0038163 | himachal pradesh | bhunter-4 | 1980 |
| 59 | IC0038164 | himachal pradesh | bhunter-5 | 1980 |
| 60 | IC038166 | himachal pradesh | Bhunter-7 | 1980 |
| 61 | IC0038620 | himachal pradesh | burua-2 | 1980 |
| 62 | IC0038188 | himachal pradesh | Butore-2 | 1980 |
| 63 | IC0038190 | himachal pradesh | butore-4 | 1980 |
| 64 | IC038291 | himachal pradesh | Chauki-10 | 1980 |
| 65 | IC0038286 | himachal pradesh | Chauki-5 | 1980 |
| 66 | IC0038107 | himachal pradesh | chemote-08 | 1980 |
| 67 | IC0038110 | himachal pradesh | Chenote-11 | 1980 |
| 68 | IC0038102 | himachal pradesh | Chenote-3 | 1980 |
| 69 | IC0038103 | himachal pradesh | Chenote-4 | 1980 |
| 70 | IC0038348 | himachal pradesh | chiri-2 | 1980 |
| 71 | IC038352 | himachal pradesh | Chiri-6 | 1980 |
| 72 | IC0038365 | himachal pradesh | churag-12 | 1980 |
| 73 | IC038356 | himachal pradesh | churag-3 | 1980 |
| 74 | IC0038359 | himachal pradesh | churag-9 | 1980 |
| 75 | IC0038334 | himachal pradesh | Dharmar-1 | 1980 |
| 76 | IC0038335 | himachal pradesh | Dharmar-2 | 1980 |
| 77 | IC0038639 | himachal pradesh | Dochi-2 | 1980 |
| 78 | IC0038127 | himachal pradesh | Dungri-5 | 1980 |
| 79 | IC0038635 | himachal pradesh | Duni-2 | 1980 |
| 80 | IC0038610 | himachal pradesh | dwarehi-6 | 1980 |
| 81 | IC0038611 | himachal pradesh | Dwarehi-7 | 1980 |
| 82 | IC0423408 | himachal pradesh |  | 2004 |
| 83 | IC0361601 | himachal pradesh |  | 2002 |
| 84 | IC0361603 | himachal pradesh |  | 2002 |
| 85 | IC0361608 | himachal pradesh |  | 2002 |
| 86 | IC0467884 | himachal pradesh |  | 2004 |
| 87 | IC0467886 | himachal pradesh |  | 2004 |
| 88 | IC0467894 | himachal pradesh |  | 2004 |
| 89 | IC0467895 | himachal pradesh |  | 2004 |
| 90 | IC0467899 | himachal pradesh |  | 2004 |
| 91 | IC0469235 | himachal pradesh |  | 1999 |
| 92 | IC0469241 | himachal pradesh |  | 1999 |
| 93 | IC0469242 | himachal pradesh |  | 1999 |
| 94 | IC0547507 | himachal pradesh |  | 2005 |
| 95 | IC0038595 | himachal pradesh | Fagu-3 | 1980 |
| 96 | IC0423410 | himachal pradesh |  | 2004 |
| 97 | IC0423537 | himachal pradesh |  | 2004 |
| 98 | IC0095352 | himachal pradesh |  | 1980 |
| 99 | IC0107845 | himachal pradesh |  | 1988 |
| 100 | IC0017940 | himachal pradesh |  | 1973 |
| 101 | IC0017926 | himachal pradesh |  | 1973 |
| 102 | IC0035696 | himachal pradesh |  | 1979 |
| 103 | IC038170 | himachal pradesh | Jatel-2 | 1980 |
| 104 | IC0038171 | himachal pradesh | Jatel-3 | 1980 |
| 105 | IC0038172 | himachal pradesh | jatel-4 | 1980 |
| 106 | IC0038395 | himachal pradesh | kaarsog-16 | 1980 |
| 107 | IC0038276 | himachal pradesh | kadai-1 | 1980 |
| 108 | IC0038278 | himachal pradesh | kadai-3 | 1980 |
| 109 | IC0038474 | himachal pradesh | kairto-1 | 1980 |
| 110 | IC0038483 | himachal pradesh | kairto-10 | 1980 |
| 111 | IC0038487 | himachal pradesh | kairto-14 | 1980 |
| 112 | IC0038480 | himachal pradesh | kairto-7 | 1980 |
| 113 | IC0038482 | himachal pradesh | kairto-9 | 1980 |
| 114 | IC0038145 | himachal pradesh | Kalbhai-3 | 1980 |
| 115 | IC0038147 | himachal pradesh | Kalbhai-5 | 1980 |
| 116 | IC0038577-5 | himachal pradesh | kalpa | 1980 |
| 117 | IC0038524 | himachal pradesh | kalpa-1 | 1980 |
| 118 | IC0038536 | himachal pradesh | kalpa-13 | 1980 |
| 119 | IC0038540 | himachal pradesh | kalpa-17 | 1980 |
| 120 | IC0038542 | himachal pradesh | kalpa-19 | 1980 |
| 121 | IC0038543 | himachal pradesh | kalpa-20 | 1980 |
| 122 | IC0038545 | himachal pradesh | kalpa-22 | 1980 |
| 123 | IC0038555 | himachal pradesh | Kalpa-32 | 1980 |
| 124 | IC0038556 | himachal pradesh | kalpa-33 | 1980 |
| 125 | IC0038559 | himachal pradesh | kalpa-36 | 1980 |
| 126 | IC0038560 | himachal pradesh | kalpa-37 | 1980 |
| 127 | IC0038562 | himachal pradesh | kalpa-39 | 1980 |
| 128 | IC0038565 | himachal pradesh | kalpa-42 | 1980 |
| 129 | IC0038569 | himachal pradesh | kalpa-46 | 1980 |
| 130 | IC0038570 | himachal pradesh | kalpa-47 | 1980 |
| 131 | IC0038528 | himachal pradesh | kalpa-5 | 1980 |
| 132 | IC0038574 | himachal pradesh | kalpa-51 | 1980 |
| 133 | IC0038575 | himachal pradesh | kalpa-52 | 1980 |
| 134 | IC0038576 | himachal pradesh | kalpa-53 | 1980 |
| 135 | IC0038577-3 | himachal pradesh | kalpa-54 | 1980 |
| 136 | IC0038579 | himachal pradesh | kalpa-61 | 1980 |
| 137 | IC0038633 | himachal pradesh | kamru-10 | 1980 |
| 138 | IC0038628 | himachal pradesh | kamru-5 | 1980 |
| 139 | IC038175 | himachal pradesh | Kanala-1 | 1980 |
| 140 | IC0038485 | himachal pradesh | Karito-12 | 1980 |
| 141 | IC0038389 | himachal pradesh | karsog-10 | 1980 |
| 142 | IC0038390 | himachal pradesh | karsog-11 | 1980 |
| 143 | IC0038391 | himachal pradesh | karsog-12 | 1980 |
| 144 | IC038392 | himachal pradesh | Karsog-13 | 1980 |
| 145 | IC038381 | himachal pradesh | Karsog-2 | 1980 |
| 146 | IC038159 | himachal pradesh | Kasol-1 | 1980 |
| 147 | IC0038235 | himachal pradesh | kelodhar-5 | 1980 |
| 148 | IC038325 | himachal pradesh | Kelodhar-5 | 1980 |
| 149 | IC0038328 | himachal pradesh | kelodhar-8 | 1980 |
| 150 | IC0038150 | himachal pradesh | Khanash-2 | 1980 |
| 151 | IC0038153 | himachal pradesh | Khanash-5 | 1980 |
| 152 | IC038157 | himachal pradesh | Khanash-9 | 1980 |
| 153 | IC0038436 | himachal pradesh | kotalu-12 | 1980 |
| 154 | IC0038431 | himachal pradesh | kotalu-7 | 1980 |
| 155 | IC0038179 | himachal pradesh | Kote-2 | 1980 |
| 156 | IC0038180 | himachal pradesh | Kote-3 | 1980 |
| 157 | IC0038185 | himachal pradesh | Kote-8 | 1980 |
| 158 | IC0038275 | himachal pradesh | koti-4 | 1980 |
| 159 | IC0035580 | himachal pradesh |  | 1989 |
| 160 | IC0093962 | himachal pradesh |  | 1987 |
| 161 | IC0094657 | himachal pradesh |  | 1988 |
| 162 | IC0094658 | himachal pradesh |  | 1988 |
| 163 | IC0094659 | himachal pradesh |  | 1988 |
| 164 | IC0095245 | himachal pradesh |  | 1988 |
| 165 | IC0095247 | himachal pradesh |  | 1988 |
| 166 | IC0095256 | himachal pradesh |  | 1988 |
| 167 | IC0095288 | himachal pradesh |  | 1980 |
| 168 | IC0095294 | himachal pradesh |  | 1962 |
| 169 | IC0095308 | himachal pradesh |  | 1962 |
| 170 | IC0095320 | himachal pradesh |  | 1985 |
| 171 | IC0095321 | himachal pradesh |  | 1962 |
| 172 | IC0095338 | himachal pradesh |  | 1985 |
| 173 | IC0095378 | himachal pradesh |  | 2001 |
| 174 | IC0095379 | himachal pradesh |  | 2001 |
| 175 | IC0095383 | himachal pradesh |  | 2001 |
| 176 | IC0095389 | himachal pradesh |  | 2001 |
| 177 | IC0095562 | himachal pradesh |  | 1989 |
| 178 | IC0095563 | himachal pradesh |  | 1989 |
| 179 | IC0095566 | himachal pradesh |  | 1989 |
| 180 | IC0095581 | himachal pradesh |  | 1989 |
| 181 | IC0095582 | himachal pradesh |  | 1989 |
| 182 | IC0095590 | himachal pradesh |  | 1989 |
| 183 | IC0095598 | himachal pradesh |  | 1989 |
| 184 | IC0095600 | himachal pradesh |  | 1989 |
| 185 | IC0105047 | himachal pradesh |  | - |
| 186 | IC0106354 | himachal pradesh |  | - |
| 187 | IC0107246 | himachal pradesh |  | 1986 |
| 188 | IC0107256 | himachal pradesh |  | 1986 |
| 189 | IC0107283 | himachal pradesh |  | 1986 |
| 190 | IC0107291 | himachal pradesh |  | 1986 |
| 191 | IC0107301 | himachal pradesh |  | 1986 |
| 192 | IC0107313 | himachal pradesh |  | 1986 |
| 193 | IC0107569 | himachal pradesh |  | - |
| 194 | IC0107578 | himachal pradesh |  | 1987 |
| 195 | IC0107615 | himachal pradesh |  | 1987 |
| 196 | IC0107617 | himachal pradesh |  | 1987 |
| 197 | IC0107696 | himachal pradesh |  | 1987 |
| 198 | IC0107826 | himachal pradesh |  | 1988 |
| 199 | IC0107829 | himachal pradesh |  | 1988 |
| 200 | IC0107838 | himachal pradesh |  | 1988 |
| 201 | IC0107847 | himachal pradesh |  | 1988 |
| 202 | IC0107848 | himachal pradesh |  | 1988 |
| 203 | IC0130220 | himachal pradesh |  | 1989 |
| 204 | IC0415220 | himachal pradesh |  | 2003 |
| 205 | IC0415222 | himachal pradesh |  | 2003 |
| 206 | IC0415250 | himachal pradesh |  | 2003 |
| 207 | IC0415254 | himachal pradesh |  | 2003 |
| 208 | IC0415262 | himachal pradesh |  | 2003 |
| 209 | IC0415264 | himachal pradesh |  | 2003 |
| 210 | IC0415268 | himachal pradesh |  | 2003 |
| 211 | IC0415269 | himachal pradesh |  | 2003 |
| 212 | IC0415318 | himachal pradesh |  | 2003 |
| 213 | IC0415418 | himachal pradesh |  | 2003 |
| 214 | IC0415429 | himachal pradesh |  | 2003 |
| 215 | IC0415448 | himachal pradesh |  | 2003 |
| 216 | IC0540832 | himachal pradesh |  | 2004 |
| 217 | IC0540835 | himachal pradesh |  | 2004 |
| 218 | IC0547370 | himachal pradesh |  | 2004 |
| 219 | IC0547375 | himachal pradesh |  | 2004 |
| 220 | IC0547382 | himachal pradesh |  | 2004 |
| 221 | IC0547387 | himachal pradesh |  | 2004 |
| 222 | IC0547393 | himachal pradesh |  | 2004 |
| 223 | IC0547395 | himachal pradesh |  | 2004 |
| 224 | IC0547397 | himachal pradesh |  | 2004 |
| 225 | IC0095638 | himachal pradesh |  | 1989 |
| 226 | IC0038048 | himachal pradesh | manikaran-1 | 1980 |
| 227 | IC0038049 | himachal pradesh | manikaran-2 | 1980 |
| 228 | IC0038052 | himachal pradesh | Mathal-1 | 1980 |
| 229 | IC0038643 | himachal pradesh | mora-2 | 1980 |
| 230 | IC0038645 | himachal pradesh | Mora-4 | 1980 |
| 231 | IC0038064 | himachal pradesh | narkanda-11 | 1980 |
| 232 | IC0038067 | himachal pradesh | narkanda-14 | 1980 |
| 233 | IC0038072 | himachal pradesh | narkanda-19 | 1980 |
| 234 | IC0038080 | himachal pradesh | Narkanda-27 | 1980 |
| 235 | IC0038058 | himachal pradesh | Narkanda-5 | 1980 |
| 236 | IC0038061 | himachal pradesh | Narkanda-8 | 1980 |
| 237 | IC0038660 | himachal pradesh | palampur-2 | 1980 |
| 238 | IC0038661 | himachal pradesh | palampur-3 | 1980 |
| 239 | IC0038592 | himachal pradesh | pangi-10 | 1980 |
| 240 | IC0038584 | himachal pradesh | pangi-2 | 1980 |
| 241 | IC0038591 | himachal pradesh | pangi-9 | 1980 |
| 242 | IC0038443 | himachal pradesh | pogna-1 | 1980 |
| 243 | IC0038454 | himachal pradesh | Pogna-12 | 1980 |
| 244 | IC0038455 | himachal pradesh | pogna-13 | 1980 |
| 245 | IC0038445 | himachal pradesh | pogna-3 | 1980 |
| 246 | IC0038450 | himachal pradesh | pogna-8 | 1980 |
| 247 | IC0243178 | himachal pradesh |  | 1988 |
| 248 | IC0243176 | himachal pradesh |  | 1998 |
| 249 | IC0038139 | himachal pradesh | Rumbee-3 | 1980 |
| 250 | IC038141 | himachal pradesh | Rumbee-5 | 1980 |
| 251 | IC0038309 | himachal pradesh | sahganaga-3 | 1980 |
| 252 | IC0038310 | himachal pradesh | sahganga-4 | 1980 |
| 253 | IC0038265 | himachal pradesh | sahran | 1980 |
| 254 | IC0038210 | himachal pradesh | sahran-10 | 1980 |
| 255 | IC0038212 | himachal pradesh | sahran-12 | 1980 |
| 256 | IC0038213 | himachal pradesh | sahran-13 | 1980 |
| 257 | IC0038218 | himachal pradesh | sahran-18 | 1980 |
| 258 | IC0038202 | himachal pradesh | sahran-2 | 1980 |
| 259 | IC0038224 | himachal pradesh | Sahran-24 | 1980 |
| 260 | IC0038226 | himachal pradesh | Sahran-26 | 1980 |
| 261 | IC0038228 | himachal pradesh | Sahran-28 | 1980 |
| 262 | IC038229 | himachal pradesh | Sahran-29 | 1980 |
| 263 | IC0038231 | himachal pradesh | sahran-31 | 1980 |
| 264 | IC0038240 | himachal pradesh | sahran-40 | 1980 |
| 265 | IC0038249 | himachal pradesh | sahran-49 | 1980 |
| 266 | IC0038205 | himachal pradesh | sahran-5 | 1980 |
| 267 | IC0038250 | himachal pradesh | sahran-50 | 1980 |
| 268 | IC0038255 | himachal pradesh | sahran-55 | 1980 |
| 269 | IC0038257 | himachal pradesh | sahran-57 | 1980 |
| 270 | IC0038206 | himachal pradesh | sahran-6 | 1980 |
| 271 | IC0038261 | himachal pradesh | sahran-61 | 1980 |
| 272 | IC0038262 | himachal pradesh | sahran-62 | 1980 |
| 273 | IC0038264 | himachal pradesh | Sahran-64 | 1980 |
| 274 | IC0038470 | himachal pradesh | sakori-1 | 1980 |
| 275 | IC0038473 | himachal pradesh | Sakori-4 | 1980 |
| 276 | IC0038505 | himachal pradesh | Sangla-17 | 1980 |
| 277 | IC0038517 | himachal pradesh | Sangla-29 | 1980 |
| 278 | IC0038494 | himachal pradesh | Sanla-6 | 1980 |
| 279 | IC0038657 | himachal pradesh | sapni-3 | 1980 |
| 280 | IC0038085 | himachal pradesh | Sarbati-2 | 1980 |
| 281 | IC0038083 | himachal pradesh | sarsai-1 | 1980 |
| 282 | IC0038097 | himachal pradesh | Seyal bagh-3 | 1980 |
| 283 | IC0038099 | himachal pradesh | Seyal bagh-5 | 1980 |
| 284 | IC0038129 | himachal pradesh | Shalbagh | 1980 |
| 285 | IC0038131 | himachal pradesh | Shalbagh-3 | 1980 |
| 286 | IC0038132 | himachal pradesh | Shalbagh-4 | 1980 |
| 287 | IC0038133 | himachal pradesh | Shalbagh-5 | 1980 |
| 288 | IC0038200 | himachal pradesh | shaleu-4 | 1980 |
| 289 | IC038366 | himachal pradesh | Sorey-1 | 1980 |
| 290 | IC0038375 | himachal pradesh | sorey-10 | 1980 |
| 291 | IC0038379 | himachal pradesh | sorey-14 | 1980 |
| 292 | IC0038367 | himachal pradesh | sorey-2 | 1980 |
| 293 | IC0038368 | himachal pradesh | sorey-3 | 1980 |
| 294 | IC038369 | himachal pradesh | Sorey-4 | 1980 |
| 295 | IC038374 | himachal pradesh | Sorey-9 | 1980 |
| 296 | IC0038621 | himachal pradesh | swang-1 | 1980 |
| 297 | IC0038403 | himachal pradesh | Teban-8 | 1980 |
| 298 | IC0017925 | himachal pradesh |  | 1973 |
| 299 | IC0017933 | himachal pradesh |  | 1973 |
| 300 | IC0017937 | himachal pradesh |  | 1973 |
| 301 | IC0017939 | himachal pradesh |  | 1973 |
| 302 | IC0017945 | himachal pradesh |  | 1973 |
| 303 | IC0017946 | himachal pradesh |  | 1973 |
| 304 | IC0017949 | himachal pradesh |  | 1973 |
| 305 | IC0017950 | himachal pradesh |  | 1973 |
| 306 | IC0017954 | himachal pradesh |  | 1973 |
| 307 | IC0017957 | himachal pradesh |  | 1973 |
| 308 | IC0018366 | himachal pradesh |  | 1973 |
| 309 | IC0018369 | himachal pradesh |  | 1973 |
| 310 | IC0035664 | himachal pradesh |  | 1979 |
| 311 | IC0035669 | himachal pradesh |  | 1979 |
| 312 | IC0035670 | himachal pradesh |  | 1979 |
| 313 | IC0035671 | himachal pradesh |  | 1979 |
| 314 | IC0035673 | himachal pradesh |  | 1979 |
| 315 | IC0035675 | himachal pradesh |  | 1979 |
| 316 | IC0035680 | himachal pradesh |  | 1979 |
| 317 | IC0035683 | himachal pradesh |  | 1979 |
| 318 | IC0035685 | himachal pradesh |  | 1979 |
| 319 | IC0035686 | himachal pradesh |  | 1979 |
| 320 | IC0035688 | himachal pradesh |  | 1979 |
| 321 | IC0035689 | himachal pradesh |  | 1979 |
| 322 | IC0035694 | himachal pradesh |  | 1979 |
| 323 | IC0035698 | himachal pradesh |  | 1979 |
| 324 | IC0035700 | himachal pradesh |  | 1979 |
| 325 | IC0035702 | himachal pradesh |  | 1979 |
| 326 | IC0035706 | himachal pradesh |  | 1979 |
| 327 | IC0035717 | himachal pradesh |  | 1979 |
| 328 | IC0035721 | himachal pradesh |  | 1979 |
| 329 | IC0035722 | himachal pradesh |  | 1979 |
| 330 | IC0035731 | himachal pradesh |  | 1979 |
| 331 | IC0035736 | himachal pradesh |  | 1979 |
| 332 | IC0035741 | himachal pradesh |  | 1979 |
| 333 | IC0035766 | himachal pradesh |  | 1979 |
| 334 | IC0038105 | himachal pradesh |  | 1980 |
| 335 | IC0038181 | himachal pradesh |  | - |
| 336 | IC0038183 | himachal pradesh |  | 1980 |
| 337 | IC0038302 | himachal pradesh |  | - |
| 338 | IC0093944 | himachal pradesh |  | 1987 |
| 339 | IC0095248 | himachal pradesh |  | 1988 |
| 340 | IC0095279 | himachal pradesh |  | 1962 |
| 341 | IC0095282 | himachal pradesh |  | 1985 |
| 342 | IC0095283 | himachal pradesh |  | 2000 |
| 343 | IC0095312 | himachal pradesh |  | - |
| 344 | IC0095337 | himachal pradesh |  | 1985 |
| 345 | IC0095346 | himachal pradesh |  | - |
| 346 | IC0095355 | himachal pradesh |  | 1985 |
| 347 | IC0095363 | himachal pradesh |  | - |
| 348 | IC0095365 | himachal pradesh |  | 1985 |
| 349 | IC0095367 | himachal pradesh |  | 1985 |
| 350 | IC0095368 | himachal pradesh |  | 1985 |
| 351 | IC0095380 | himachal pradesh |  | 2001 |
| 352 | IC0095382 | himachal pradesh |  | - |
| 353 | IC0095386 | himachal pradesh |  | 2001 |
| 354 | IC0095391 | himachal pradesh |  | 2001 |
| 355 | IC0095631 | himachal pradesh |  | - |
| 356 | IC0107007 | himachal pradesh |  | 1986 |
| 357 | IC0107096 | himachal pradesh |  | 1989 |
| 358 | IC0107144 | himachal pradesh |  | 1986 |
| 359 | IC0107233 | himachal pradesh |  | 1986 |
| 360 | IC0107276 | himachal pradesh |  | 1986 |
| 361 | IC0107334 | himachal pradesh |  | 1986 |
| 362 | IC0107350 | himachal pradesh |  | 1986 |
| 363 | IC0107531 | himachal pradesh |  | 1987 |
| 364 | IC0107626 | himachal pradesh |  | 1987 |
| 365 | IC0107688 | himachal pradesh |  | 1987 |
| 366 | IC0107832 | himachal pradesh |  | 1988 |
| 367 | IC0107835 | himachal pradesh |  | 1988 |
| 368 | IC0107843 | himachal pradesh |  | 1988 |
| 369 | IC0108419 | himachal pradesh |  | 1985 |
| 370 | IC0108426 | himachal pradesh |  | 1985 |
| 371 | IC0108427 | himachal pradesh |  | 1985 |
| 372 | IC0108428 | himachal pradesh |  | 1985 |
| 373 | IC0108429 | himachal pradesh |  | 1985 |
| 374 | IC0108431 | himachal pradesh |  | 1985 |
| 375 | IC0258400 | himachal pradesh |  | 1999 |
| 376 | IC0265926 | himachal pradesh |  | 2000 |
| 377 | IC0274448 | himachal pradesh |  | 2000 |
| 378 | IC0274450 | himachal pradesh |  | 2000 |
| 379 | IC0274462 | himachal pradesh |  | 2000 |
| 380 | IC0274472 | himachal pradesh |  | 2000 |
| 381 | IC0274473 | himachal pradesh |  | 2000 |
| 382 | IC0278913 | himachal pradesh |  | 2000 |
| 383 | IC0278919 | himachal pradesh |  | 2000 |
| 384 | IC0279962 | himachal pradesh |  | 2000 |
| 385 | IC0279968 | himachal pradesh |  | 2000 |
| 386 | IC0279970 | himachal pradesh |  | 2000 |
| 387 | IC0279973 | himachal pradesh |  | 2000 |
| 388 | IC0310049 | himachal pradesh |  | 2000 |
| 389 | IC0313258 | himachal pradesh |  | 2000 |
| 390 | IC0313263 | himachal pradesh |  | 2000 |
| 391 | IC0313271 | himachal pradesh |  | 2000 |
| 392 | IC0313273 | himachal pradesh |  | 2000 |
| 393 | IC0313274 | himachal pradesh |  | 2000 |
| 394 | IC0329492 | himachal pradesh |  | 2001 |
| 395 | IC0329513 | himachal pradesh |  | 2001 |
| 396 | IC0329514 | himachal pradesh |  | 2001 |
| 397 | IC0329550 | himachal pradesh |  | 2001 |
| 398 | IC0329587 | himachal pradesh |  | 2001 |
| 399 | IC038426 | himachal pradesh |  | 1980 |
| 400 | IC038510 | himachal pradesh |  | 1980 |
| 401 | IC0423393 | himachal pradesh |  | 2004 |
| 402 | IC0423551 | himachal pradesh |  | 2004 |
| 403 | IC0506493 | himachal pradesh |  | - |
| 404 | IC0506505 | himachal pradesh |  | - |
| 405 | IC423462 | himachal pradesh |  | 2004 |
| 406 | IC0616908 | jammu and kashmir |  | 2014 |
| 407 | IC0021789 | madhya pradesh |  | 1974 |
| 408 | IC0095534 | madhya pradesh |  | - |
| 409 | IC0042000 | madhya pradesh |  | 1981 |
| 410 | IC0041988 | madhya pradesh |  | 1981 |
| 411 | IC0042002 | madhya pradesh |  | 1981 |
| 412 | IC0042006 | madhya pradesh |  | 1981 |
| 413 | IC0021802-1 | madhya pradesh |  | 1974 |
| 414 | IC0021808 | madhya pradesh |  | 1974 |
| 415 | IC0021944 | madhya pradesh |  | 1974 |
| 416 | IC0041999 | madhya pradesh |  | 1981 |
| 417 | IC0095628 | madhya pradesh |  | - |
| 418 | IC0120585 | madhya pradesh |  | - |
| 419 | IC0035438 | maharashtra |  | 1979 |
| 420 | IC0035407 | maharashtra |  | 1979 |
| 421 | IC0550693 | maharashtra |  | 2006 |
| 422 | IC0618379 | maharashtra |  | 2015 |
| 423 | IC0095556 | maharashtra |  | 1986 |
| 424 | IC0095260 | maharashtra |  | - |
| 425 | IC0095479 | maharashtra |  | - |
| 426 | IC0095514 | maharashtra |  | - |
| 427 | IC0095531 | maharashtra |  | - |
| 428 | IC0110306 | maharashtra |  | - |
| 429 | IC0042011 | maharashtra |  | 1981 |
| 430 | IC0041987 | maharashtra |  | 1981 |
| 431 | IC0035370 | maharashtra |  | 1979 |
| 432 | IC0035496 | maharashtra |  | 1979 |
| 433 | IC0032179 | maharashtra |  | 1978 |
| 434 | IC0035362 | maharashtra |  | 1979 |
| 435 | IC0035363 | maharashtra |  | 1979 |
| 436 | IC0035366 | maharashtra |  | 1979 |
| 437 | IC0035371 | maharashtra |  | 1979 |
| 438 | IC0035372 | maharashtra |  | 1979 |
| 439 | IC0035373 | maharashtra |  | 1979 |
| 440 | IC0035378 | maharashtra |  | 1979 |
| 441 | IC0035386 | maharashtra |  | 1979 |
| 442 | IC0035387 | maharashtra |  | 1979 |
| 443 | IC0035391 | maharashtra |  | 1979 |
| 444 | IC0035393 | maharashtra |  | 1979 |
| 445 | IC0035394 | maharashtra |  | 1979 |
| 446 | IC0035398 | maharashtra |  | 1979 |
| 447 | IC0035399 | maharashtra |  | 1979 |
| 448 | IC0035401 | maharashtra |  | 1979 |
| 449 | IC0035403 | maharashtra |  | 1979 |
| 450 | IC0035406 | maharashtra |  | 1979 |
| 451 | IC0035409 | maharashtra |  | 1979 |
| 452 | IC0035412 | maharashtra |  | 1979 |
| 453 | IC0035414 | maharashtra |  | 1979 |
| 454 | IC0035415 | maharashtra |  | 1979 |
| 455 | IC0035419 | maharashtra |  | 1979 |
| 456 | IC0035420 | maharashtra |  | 1979 |
| 457 | IC0035422 | maharashtra |  | 1979 |
| 458 | IC0035425 | maharashtra |  | 1979 |
| 459 | IC0035429 | maharashtra |  | 1979 |
| 460 | IC0035430 | maharashtra |  | 1979 |
| 461 | IC0035431 | maharashtra |  | 1979 |
| 462 | IC0035434 | maharashtra |  | 1979 |
| 463 | IC0035436 | maharashtra |  | 1979 |
| 464 | IC0035443 | maharashtra |  | 1979 |
| 465 | IC0035444 | maharashtra |  | 1979 |
| 466 | IC0035445 | maharashtra |  | 1979 |
| 467 | IC0035449 | maharashtra |  | 1979 |
| 468 | IC0035450 | maharashtra |  | 1979 |
| 469 | IC0035459 | maharashtra |  | 1979 |
| 470 | IC0035464 | maharashtra |  | 1979 |
| 471 | IC0035465 | maharashtra |  | 1979 |
| 472 | IC0035466 | maharashtra |  | 1979 |
| 473 | IC0035476 | maharashtra |  | 1979 |
| 474 | IC0035478 | maharashtra |  | 1979 |
| 475 | IC0035485 | maharashtra |  | 1979 |
| 476 | IC0035487 | maharashtra |  | 1979 |
| 477 | IC0035489 | maharashtra |  | 1979 |
| 478 | IC0035491 | maharashtra |  | 1979 |
| 479 | IC0035498 | maharashtra |  | 1979 |
| 480 | IC0035499 | maharashtra |  | 1979 |
| 481 | IC0035503 | maharashtra |  | 1979 |
| 482 | IC0035506 | maharashtra |  | 1979 |
| 483 | IC0055144 | maharashtra |  | 1982 |
| 484 | IC0055149 | maharashtra |  | 1982 |
| 485 | IC0095643 | maharashtra |  | - |
| 486 | IC0120588 | maharashtra |  | - |
| 487 | IC0415675 | maharashtra |  | 2003 |
| 488 | IC-55143 | maharashtra |  | 1982 |
| 489 | IC0281919 | odisha |  | 2000 |
| 490 | IC0281924 | odisha |  | 2000 |
| 491 | IC0281925 | odisha |  | 2000 |
| 492 | IC0281936 | odisha |  | 2000 |
| 493 | IC0281928 | odisha |  | 2000 |
| 494 | IC0095639 | odisha |  | - |
| 495 | IC0281930 | odisha |  | 2000 |
| 496 | IC0627440 | rajasthan |  | 2018 |
| 497 | IC0037320 | sikkim |  | 1980 |
| 498 | IC0042258 | uttar pradesh |  | 1981 |
| 499 | IC0042264 | uttar pradesh |  | 1981 |
| 500 | IC0038041 | uttar pradesh | hamirpur-1 | 1980 |
| 501 | IC0038042 | uttar pradesh | hamirpur-2 | 1980 |
| 502 | IC0042332 | uttar pradesh |  | 1981 |
| 503 | IC0095601 | uttar pradesh |  | 1989 |
| 504 | IC0016636 | uttar pradesh |  | - |
| 505 | IC0528321 | uttar pradesh |  | 2005 |
| 506 | IC0528349 | uttar pradesh |  | 2005 |
| 507 | IC0528306 | uttar pradesh |  | 2005 |
| 508 | IC0042321 | uttar pradesh |  | 1981 |
| 509 | IC0047438 | uttar pradesh |  | 1981 |
| 510 | IC0448717 | uttarakhand |  | 2004 |
| 511 | IC0448720 | uttarakhand |  | 2004 |
| 512 | IC0448770 | uttarakhand |  | 2004 |
| 513 | IC0444147 | uttarakhand |  | 2004 |
| 514 | IC0042290-16 | uttarakhand |  | 1981 |
| 515 | IC0042290-20 | uttarakhand |  | 1981 |
| 516 | IC0042293-16 | uttarakhand |  | 1981 |
| 517 | IC0042293-8 | uttarakhand |  | 1981 |
| 518 | IC0042309-5 | uttarakhand |  | 1981 |
| 519 | IC0042309 | uttarakhand |  | 1981 |
| 520 | IC0095409 | uttarakhand |  | 1985 |
| 521 | IC0095544 | uttarakhand |  | 1988 |
| 522 | IC0553952 | uttarakhand |  | 2007 |
| 523 | IC0553954 | uttarakhand |  | 2007 |
| 524 | IC0553959 | uttarakhand |  | 2007 |
| 525 | IC0553961 | uttarakhand |  | 2007 |
| 526 | IC0553963 | uttarakhand |  | 2007 |
| 527 | IC0553964 | uttarakhand |  | 2007 |
| 528 | IC0553968 | uttarakhand |  | 2007 |
| 529 | IC0553973 | uttarakhand |  | 2007 |
| 530 | IC0553974 | uttarakhand |  | 2007 |
| 531 | IC0553976 | uttarakhand |  | 2007 |
| 532 | IC0553981 | uttarakhand |  | 2007 |
| 533 | IC0553982 | uttarakhand |  | 2007 |
| 534 | IC0553983 | uttarakhand |  | 2007 |
| 535 | IC0553987 | uttarakhand |  | 2007 |
| 536 | IC0553988 | uttarakhand |  | 2007 |
| 537 | IC0553993 | uttarakhand |  | 2007 |
| 538 | IC0553994 | uttarakhand |  | 2007 |
| 539 | IC0553995 | uttarakhand |  | 2007 |
| 540 | IC0553998 | uttarakhand |  | 2007 |
| 541 | IC0554001 | uttarakhand |  | 2007 |
| 542 | IC0554002 | uttarakhand |  | 2007 |
| 543 | IC0554003 | uttarakhand |  | 2007 |
| 544 | IC0554007 | uttarakhand |  | 2007 |
| 545 | IC0554008 | uttarakhand |  | 2007 |
| 546 | IC0554010 | uttarakhand |  | 2007 |
| 547 | IC0554012 | uttarakhand |  | 2007 |
| 548 | IC0554017 | uttarakhand |  | 2007 |
| 549 | IC0554018 | uttarakhand |  | 2007 |
| 550 | IC0554019 | uttarakhand |  | 2007 |
| 551 | IC0554022 | uttarakhand |  | 2007 |
| 552 | IC0554026 | uttarakhand |  | 2007 |
| 553 | IC0554038 | uttarakhand |  | 2007 |
| 554 | IC0554050 | uttarakhand |  | 2007 |
| 555 | IC0554051 | uttarakhand |  | 2007 |
| 556 | IC0554053 | uttarakhand |  | 2007 |
| 557 | IC0554054 | uttarakhand |  | 2007 |
| 558 | IC0554056 | uttarakhand |  | 2007 |
| 559 | IC0554057 | uttarakhand |  | 2007 |
| 560 | IC0554062 | uttarakhand |  | 2007 |
| 561 | IC0554065 | uttarakhand |  | 2007 |
| 562 | IC0554066 | uttarakhand |  | 2007 |
| 563 | IC0554067 | uttarakhand |  | 2007 |
| 564 | IC0554069 | uttarakhand |  | 2007 |
| 565 | IC0554073 | uttarakhand |  | 2007 |
| 566 | IC0554077 | uttarakhand |  | 2007 |
| 567 | IC0554078 | uttarakhand |  | 2007 |
| 568 | IC0554079 | uttarakhand |  | 2007 |
| 569 | IC0554082 | uttarakhand |  | 2007 |
| 570 | IC0554091 | uttarakhand |  | 2007 |
| 571 | IC0554094 | uttarakhand |  | 2007 |
| 572 | IC0554097 | uttarakhand |  | 2007 |
| 573 | IC0554101 | uttarakhand |  | 2007 |
| 574 | IC0554103 | uttarakhand |  | 2007 |
| 575 | IC0554108 | uttarakhand |  | 2007 |
| 576 | IC0554116 | uttarakhand |  | 2007 |
| 577 | IC0554117 | uttarakhand |  | 2007 |
| 578 | IC0554119 | uttarakhand |  | 2007 |
| 579 | IC0554120 | uttarakhand |  | 2007 |
| 580 | IC0554123 | uttarakhand |  | 2007 |
| 581 | IC0554125 | uttarakhand |  | 2007 |
| 582 | IC0554126 | uttarakhand |  | 2007 |
| 583 | IC0554128 | uttarakhand |  | 2007 |
| 584 | IC0554130 | uttarakhand |  | 2007 |
| 585 | IC0554132 | uttarakhand |  | 2007 |
| 586 | IC0554135 | uttarakhand |  | 2007 |
| 587 | IC0554137 | uttarakhand |  | 2007 |
| 588 | IC0554141 | uttarakhand |  | 2007 |
| 589 | IC0554146 | uttarakhand |  | 2007 |
| 590 | IC0554148 | uttarakhand |  | 2007 |
| 591 | IC0554150 | uttarakhand |  | 2007 |
| 592 | IC0554152 | uttarakhand |  | 2007 |
| 593 | IC0554158 | uttarakhand |  | 2007 |
| 594 | IC0554160 | uttarakhand |  | 2007 |
| 595 | IC0554161 | uttarakhand |  | 2007 |
| 596 | IC0554163 | uttarakhand |  | 2007 |
| 597 | IC0554165 | uttarakhand |  | 2007 |
| 598 | IC0554168 | uttarakhand |  | 2007 |
| 599 | IC0554173 | uttarakhand |  | 2007 |
| 600 | IC0554175 | uttarakhand |  | 2007 |
| 601 | IC0120851 | uttarakhand |  | 1990 |
| 602 | IC0016638 | uttarakhand |  | 1972 |
| 603 | IC0042254 | uttarakhand |  | 1981 |
| 604 | IC0042254-14 | uttarakhand |  | 1981 |
| 605 | IC0042254-15 | uttarakhand |  | 1981 |
| 606 | IC0042254-8 | uttarakhand |  | 1981 |
| 607 | IC0042267 | uttarakhand |  | 1981 |
| 608 | IC0042267-4 | uttarakhand |  | 1981 |
| 609 | IC0042272 | uttarakhand |  | 1981 |
| 610 | IC0042275-2 | uttarakhand |  | 1981 |
| 611 | IC0042277 | uttarakhand |  | 1981 |
| 612 | IC0042277-2 | uttarakhand |  | 1981 |
| 613 | IC0042277-9 | uttarakhand |  | 1981 |
| 614 | IC0042279-2 | uttarakhand |  | 1981 |
| 615 | IC0042279-6 | uttarakhand |  | 1981 |
| 616 | IC0042279-8 | uttarakhand |  | 1981 |
| 617 | IC0042281-11 | uttarakhand |  | 1981 |
| 618 | IC0042283-4 | uttarakhand |  | 1981 |
| 619 | IC0042283-8 | uttarakhand |  | 1981 |
| 620 | IC0042284-10 | uttarakhand |  | 1981 |
| 621 | IC0042284-17 | uttarakhand |  | 1981 |
| 622 | IC0042292 | uttarakhand |  | 1981 |
| 623 | IC0042292-11 | uttarakhand |  | 1981 |
| 624 | IC0042295 | uttarakhand |  | 1981 |
| 625 | IC0042310-2 | uttarakhand |  | 1981 |
| 626 | IC0042313-1 | uttarakhand |  | 1981 |
| 627 | IC0042316-1 | uttarakhand |  | 1981 |
| 628 | IC0042344 | uttarakhand |  | 1981 |
| 629 | IC0042345 | uttarakhand |  | 1981 |
| 630 | IC0042352 | uttarakhand |  | 1981 |
| 631 | IC0042358 | uttarakhand |  | 1981 |
| 632 | IC0081702 | uttarakhand |  | 1987 |
| 633 | IC0081708 | uttarakhand |  | 1985 |
| 634 | IC0081710 | uttarakhand |  | 1985 |
| 635 | IC0095426 | uttarakhand |  | - |
| 636 | IC0095433 | uttarakhand |  | - |
| 637 | IC0095542 | uttarakhand |  | 1988 |
| 638 | IC0107840 | uttarakhand |  | 1988 |
| 639 | IC0448618 | uttarakhand |  | 2004 |
| 640 | IC0448619 | uttarakhand |  | 2004 |
| 641 | IC0448620 | uttarakhand |  | 2004 |
| 642 | IC0448627 | uttarakhand |  | 2004 |
| 643 | IC0448628 | uttarakhand |  | 2004 |
| 644 | IC0448632 | uttarakhand |  | 2004 |
| 645 | IC0448633 | uttarakhand |  | 2004 |
| 646 | IC0448635 | uttarakhand |  | 2004 |
| 647 | IC0448639 | uttarakhand |  | 2004 |
| 648 | IC0448641 | uttarakhand |  | 2004 |
| 649 | IC0448642 | uttarakhand |  | 2004 |
| 650 | IC0448643 | uttarakhand |  | 2004 |
| 651 | IC0448645 | uttarakhand |  | 2004 |
| 652 | IC0448646 | uttarakhand |  | 2004 |
| 653 | IC0448649 | uttarakhand |  | 2004 |
| 654 | IC0448652 | uttarakhand |  | 2004 |
| 655 | IC0448653 | uttarakhand |  | 2004 |
| 656 | IC0448655 | uttarakhand |  | 2004 |
| 657 | IC0448658 | uttarakhand |  | 2004 |
| 658 | IC0448660 | uttarakhand |  | 2004 |
| 659 | IC0448663 | uttarakhand |  | 2004 |
| 660 | IC0448665 | uttarakhand |  | 2004 |
| 661 | IC0448666 | uttarakhand |  | 2004 |
| 662 | IC0448672 | uttarakhand |  | 2004 |
| 663 | IC0448673 | uttarakhand |  | 2004 |
| 664 | IC0448675 | uttarakhand |  | 2004 |
| 665 | IC0448685 | uttarakhand |  | 2004 |
| 666 | IC0448688 | uttarakhand |  | 2004 |
| 667 | IC0448689 | uttarakhand |  | 2004 |
| 668 | IC0448693 | uttarakhand |  | 2004 |
| 669 | IC0448694 | uttarakhand |  | 2004 |
| 670 | IC0448696 | uttarakhand |  | 2004 |
| 671 | IC0448700 | uttarakhand |  | 2004 |
| 672 | IC0448701 | uttarakhand |  | 2004 |
| 673 | IC0448702 | uttarakhand |  | 2004 |
| 674 | IC0448713 | uttarakhand |  | 2004 |
| 675 | IC0448714 | uttarakhand |  | 2004 |
| 676 | IC0448716 | uttarakhand |  | 2004 |
| 677 | IC0448719 | uttarakhand |  | 2004 |
| 678 | IC0448723 | uttarakhand |  | 2004 |
| 679 | IC0448724 | uttarakhand |  | 2004 |
| 680 | IC0448725 | uttarakhand |  | 2004 |
| 681 | IC0448726 | uttarakhand |  | 2004 |
| 682 | IC0448729 | uttarakhand |  | 2004 |
| 683 | IC0448733 | uttarakhand |  | 2004 |
| 684 | IC0448734 | uttarakhand |  | 2004 |
| 685 | IC0448735 | uttarakhand |  | 2004 |
| 686 | IC0448740 | uttarakhand |  | 2004 |
| 687 | IC0448741 | uttarakhand |  | 2004 |
| 688 | IC0448747 | uttarakhand |  | 2004 |
| 689 | IC0448749 | uttarakhand |  | 2004 |
| 690 | IC0448750 | uttarakhand |  | 2004 |
| 691 | IC0448751 | uttarakhand |  | 2004 |
| 692 | IC0448755 | uttarakhand |  | 2004 |
| 693 | IC0448758 | uttarakhand |  | 2004 |
| 694 | IC0448759 | uttarakhand |  | 2004 |
| 695 | IC0448761 | uttarakhand |  | 2004 |
| 696 | IC0448763 | uttarakhand |  | 2004 |
| 697 | IC0448766 | uttarakhand |  | 2004 |
| 698 | IC0448769 | uttarakhand |  | 2004 |
| 699 | IC0448772 | uttarakhand |  | 2004 |
| 700 | IC0448782 | uttarakhand |  | 2004 |
| 701 | IC0448783 | uttarakhand |  | 2004 |
| 702 | IC0448786 | uttarakhand |  | 2004 |
| 703 | IC0448787 | uttarakhand |  | 2004 |
| 704 | IC0448788 | uttarakhand |  | 2004 |
| 705 | IC0448790 | uttarakhand |  | 2004 |
| 706 | IC0448791 | uttarakhand |  | 2004 |
| 707 | IC0448792 | uttarakhand |  | 2004 |
| 708 | IC0506602 | uttarakhand |  | - |
| 709 | IC0554080 | uttarakhand |  | 2007 |
| 710 | IC-81701-A | uttarakhand |  | 1987 |
| 711 | IC0094652 | andhra pradesh |  | 1988 |
| 712 | IC0025788 | arunachal pradesh | bari chulai | 1976 |
| 713 | IC0412770 | arunachal pradesh |  | 2003 |
| 714 | IC0622172 | arunachal pradesh |  | 2016 |
| 715 | IC0331578 | bihar |  | 2001 |
| 716 | IC0021796-A | chattisgarh |  | 1974 |
| 717 | IC0021799 | chattisgarh |  | 1974 |
| 718 | IC0021923 | chattisgarh |  | 1974 |
| 719 | IC0047434 | chattisgarh |  | 1980 |
| 720 | EC0277970 | exotic |  | - |
| 721 | EC0519523 | exotic |  | - |
| 722 | EC0519555 | exotic |  | - |
| 723 | EC0289394 | exotic |  | 1996 |
| 724 | EC0519517 | exotic |  | 2005 |
| 725 | EC0519535 | exotic |  | 2005 |
| 726 | EC0519546 | exotic |  | 2005 |
| 727 | EC0524457 | exotic |  | 2005 |
| 728 | EC0359417 | exotic |  | - |
| 729 | EC0014586 | exotic |  | - |
| 730 | EC0120050 | exotic |  | - |
| 731 | EC0146547 | exotic |  | - |
| 732 | EC0169606 | exotic |  | - |
| 733 | EC0169611 | exotic |  | - |
| 734 | EC0169615 | exotic |  | - |
| 735 | EC0169622 | exotic |  | - |
| 736 | EC0169625 | exotic |  | - |
| 737 | EC0198117 | exotic |  | - |
| 738 | EC0198135 | exotic |  | - |
| 739 | EC0198136 | exotic |  | - |
| 740 | EC0223640 | exotic |  | - |
| 741 | EC0223642 | exotic |  | - |
| 742 | EC0223663 | exotic |  | - |
| 743 | EC0322994 | exotic |  | - |
| 744 | EC0359409 | exotic |  | 2003 |
| 745 | EC0359421 | exotic |  | - |
| 746 | EC0359431 | exotic |  | - |
| 747 | EC0359435 | exotic |  | - |
| 748 | EC0359436 | exotic |  | - |
| 749 | EC0377618 | exotic |  | - |
| 750 | EC0387002 | exotic |  | - |
| 751 | EC0387012 | exotic |  | - |
| 752 | EC0387022 | exotic |  |  |
| 753 | EC0519519 | exotic |  | - |
| 754 | EC0519524 | exotic |  | - |
| 755 | EC0519528 | exotic |  | - |
| 756 | EC0519540 | exotic |  | - |
| 757 | EC0519542 | exotic |  | - |
| 758 | EC0519547 | exotic |  | - |
| 759 | EC0519550 | exotic |  | - |
| 760 | EC0519554 | exotic |  | - |
| 761 | EC0524458 | exotic |  | - |
| 762 | IC0035614 | gujarat |  | 1979 |
| 763 | IC0035624 | gujarat |  | 1979 |
| 764 | IC0394085 | gujarat |  | 2003 |
| 765 | IC0394111 | gujarat |  | 2003 |
| 766 | IC0035560 | gujarat |  | 1979 |
| 767 | IC0035562 | gujarat |  | 1979 |
| 768 | IC0035563 | gujarat |  | 1979 |
| 769 | IC0035564 | gujarat |  | 1979 |
| 770 | IC0035565 | gujarat |  | 1979 |
| 771 | IC0035566 | gujarat |  | 1979 |
| 772 | IC0035569 | gujarat |  | 1979 |
| 773 | IC0035579 | gujarat |  | 1979 |
| 774 | IC0035582 | gujarat |  | 1979 |
| 775 | IC0035583 | gujarat |  | 1979 |
| 776 | IC0035587 | gujarat |  | 1979 |
| 777 | IC0035588 | gujarat |  | 1979 |
| 778 | IC0035590 | gujarat |  | 1979 |
| 779 | IC0035592 | gujarat |  | 1979 |
| 780 | IC0035593 | gujarat |  | 1979 |
| 781 | IC0035594 | gujarat |  | 1979 |
| 782 | IC0035599 | gujarat |  | 1979 |
| 783 | IC0035603 | gujarat |  | 1979 |
| 784 | IC0035607 | gujarat |  | 1979 |
| 785 | IC0035611 | gujarat |  | 1979 |
| 786 | IC0035613 | gujarat |  | 1979 |
| 787 | IC0035616 | gujarat |  | 1979 |
| 788 | IC0035618 | gujarat |  | 1979 |
| 789 | IC0035619 | gujarat |  | 1979 |
| 790 | IC0035623 | gujarat |  | 1979 |
| 791 | IC0035633 | gujarat |  | 1979 |
| 792 | IC0035634 | gujarat |  | 1979 |
| 793 | IC0035638 | gujarat |  | 1979 |
| 794 | IC0035640 | gujarat |  | 1979 |
| 795 | IC0035641 | gujarat |  | 1979 |
| 796 | IC0035646 | gujarat |  | 1979 |
| 797 | IC0035649 | gujarat |  | 1979 |
| 798 | IC0035655 | gujarat |  | 1979 |
| 799 | IC0035656 | gujarat |  | 1979 |
| 800 | IC0035658 | gujarat |  | 1979 |
| 801 | IC0035659 | gujarat |  | 1979 |
| 802 | IC0035780 | gujarat |  | 1979 |
| 803 | IC0038046 | haryana | Kaithal-1 | 1980 |
| 804 | IC0625051 | jammu and kashmir |  | 2017 |
| 805 | IC469539 | karnataka |  | 2004 |
| 806 | IC469674 | Karnataka |  | 2004 |
| 807 | IC0038187 | kerala | kote-4 | 1980 |
| 808 | IC0095453 | kerala |  | - |
| 809 | IC0041992 | madhya pradesh |  | 1981 |
| 810 | IC0021802 | madhya pradesh |  | 1974 |
| 811 | IC0021802-A | madhya pradesh |  | 1974 |
| 812 | IC0021805 | madhya pradesh |  | 1974 |
| 813 | IC0021925 | madhya pradesh |  | 1974 |
| 814 | IC0095642 | madhya pradesh |  | - |
| 815 | IC0035441 | maharashtra |  | 1979 |
| 816 | IC0035453 | maharashtra |  | 1979 |
| 817 | IC0625391 | nagaland |  | 2017 |
| 818 | IC0618539 | nagaland |  | 2016 |
| 819 | IC0281921 | odisha |  | 2000 |
| 820 | IC0281942 | odisha |  | 2000 |
| 821 | IC0281922 | odisha |  | 2000 |
| 822 | IC0281933 | odisha |  | 2000 |
| 823 | Annapurna1 | others |  | - |
| 824 | Annapurna2 | others |  | - |
| 825 | IC0005626 | others |  | 1958 |
| 826 | IC0007930 | others |  | 1960 |
| 827 | IC0007931 | others |  | 1960 |
| 828 | IC0007941 | others |  | 1960 |
| 829 | IC0038125 | others |  | 2005 |
| 830 | IC0042090-17 | others |  | - |
| 831 | IC0065548 | others |  | - |
| 832 | IC0093942 | others |  | 2000 |
| 833 | IC0095204 | others |  | 1989 |
| 834 | IC0095257 | others |  | - |
| 835 | IC0095259 | others |  | - |
| 836 | IC0095265 | others |  | - |
| 837 | IC0095266 | others |  | - |
| 838 | IC0095305 | others |  | - |
| 839 | IC0095318 | others |  | - |
| 840 | IC0095474 | others |  | - |
| 841 | IC0095475 | others |  | - |
| 842 | IC0095476 | others |  | - |
| 843 | IC0095482 | others |  | - |
| 844 | IC0095483 | others |  | - |
| 845 | IC0095484 | others |  | - |
| 846 | IC0095487 | others |  | - |
| 847 | IC0095489 | others |  | - |
| 848 | IC0095502 | others |  | - |
| 849 | IC0095508 | others |  | - |
| 850 | IC0095533 | others |  | - |
| 851 | IC0095537 | others |  | - |
| 852 | IC0095538 | others |  | - |
| 853 | IC0095610 | others |  | - |
| 854 | IC0095617 | others |  | - |
| 855 | IC0110269 | others |  | - |
| 856 | IC0110271 | others |  | 1988 |
| 857 | IC0120567 | others |  | - |
| 858 | IC0120568 | others |  | - |
| 859 | IC0120569 | others |  | - |
| 860 | IC0120573 | others |  | - |
| 861 | IC0120599 | others |  | - |
| 862 | IC0120603 | others |  | - |
| 863 | IC0120606 | others |  | - |
| 864 | IC0120608 | others |  | - |
| 865 | IC0120613 | others |  | - |
| 866 | IC0120622 | others |  | - |
| 867 | IC0120629 | others |  | - |
| 868 | IC0120633 | others |  | - |
| 869 | IC0120640 | others |  | - |
| 870 | IC0120643 | others |  | - |
| 871 | IC0120653 | others |  | - |
| 872 | IC0120655 | others |  | - |
| 873 | IC0120676 | others |  | - |
| 874 | IC0120679 | others |  | - |
| 875 | IC0120691 | others |  | - |
| 876 | IC0120693 | others |  | - |
| 877 | IC0120694 | others |  | - |
| 878 | IC0120696 | others |  | - |
| 879 | IC0506595 | others |  | - |
| 880 | IC-35791 | others |  | - |
| 881 | IC415271 | others |  | 1980 |
| 882 | Suvarna | others |  | - |
| 883 | IC415592 | rajasthan |  | 2003 |
| 884 | IC0586032 | rajasthan |  | 2005 |
| 885 | IC415591 | rajasthan |  | 2003 |
| 886 | IC0037323 | sikkim |  | 1980 |
| 887 | IC0095449 | tamil nadu |  | - |
| 888 | IC0095450 | tamil nadu |  | - |
| 889 | IC0095455 | tamil nadu |  | - |
| 890 | IC0095456 | tamil nadu |  | - |
| 891 | IC0095459 | tamil nadu |  | - |
| 892 | IC0095460 | tamil nadu |  | - |
| 893 | IC0095463 | tamil nadu |  | - |
| 894 | IC0095464 | tamil nadu |  | - |
| 895 | IC0095465 | tamil nadu |  | - |
| 896 | IC0095466 | tamil nadu |  | - |
| 897 | IC0249597 | telangana |  | 1999 |
| 898 | IC0042264-14 | uttar pradesh |  | 1981 |
| 899 | IC0042264-6 | uttar pradesh |  | 1981 |
| 900 | IC0042264-X | uttar pradesh |  | 1981 |
| 901 | IC0095503 | uttar pradesh |  | - |
| 902 | IC0042316-11 | uttar pradesh |  | 1981 |
| 903 | IC0016639 | uttar pradesh |  | 1972 |
| 904 | IC0042258-1 | uttar pradesh |  | 1981 |
| 905 | IC0042258-10 | uttar pradesh |  | 1981 |
| 906 | IC0042258-11 | uttar pradesh |  | 1981 |
| 907 | IC0042258-13 | uttar pradesh |  | 1981 |
| 908 | IC0042261-1 | uttar pradesh |  | 1981 |
| 909 | IC0042262-8 | uttar pradesh |  | 1981 |
| 910 | IC0042301 | uttar pradesh |  | 1981 |
| 911 | IC0042315-3 | uttar pradesh |  | 1981 |
| 912 | IC0042315-5 | uttar pradesh |  | 1981 |
| 913 | IC0042315-7 | uttar pradesh |  | - |
| 914 | IC0042315-8 | uttar pradesh |  | 1981 |
| 915 | IC0042319-1 | uttar pradesh |  | 1981 |
| 916 | IC0095541 | uttar pradesh |  | - |
| 917 | IC0095608 | uttar pradesh |  | - |

**Supplementary Table 2.** Distribution of SNP loci in the AmahySNP array in the amaranth genome.

| **Scaffold** | **No. of genes** | **Genic SNPs** | **Intergenic SNPs** | **Exons** | **Introns** | **5'UTR** | **3'UTR** | **Total SNPs** |
| --- | --- | --- | --- | --- | --- | --- | --- | --- |
| Scaffold_1 | 924 | 3673 | 2898 | 696 | 2710 | 93 | 174 | 6571 |
| Scaffold_2 | 1020 | 3879 | 3198 | 788 | 2743 | 135 | 213 | 7077 |
| Scaffold_3 | 586 | 2210 | 2431 | 416 | 1626 | 54 | 114 | 4641 |
| Scaffold_4 | 554 | 2296 | 1349 | 422 | 1689 | 65 | 120 | 3645 |
| Scaffold_5 | 766 | 3405 | 2911 | 652 | 2423 | 123 | 207 | 6316 |
| Scaffold_6 | 795 | 3514 | 2937 | 659 | 2578 | 86 | 191 | 6451 |
| Scaffold_7 | 227 | 770 | 701 | 133 | 587 | 23 | 27 | 1471 |
| Scaffold_8 | 588 | 2313 | 2161 | 474 | 1637 | 67 | 135 | 4474 |
| Scaffold_9 | 700 | 2778 | 2625 | 528 | 2038 | 80 | 132 | 5403 |
| Scaffold_10 | 515 | 2292 | 1802 | 382 | 1745 | 60 | 105 | 4094 |
| Scaffold_11 | 514 | 1962 | 1541 | 419 | 1374 | 68 | 101 | 3503 |
| Scaffold_12 | 183 | 352 | 331 | 66 | 251 | 17 | 18 | 683 |
| Scaffold_13 | 561 | 2483 | 1433 | 435 | 1838 | 67 | 143 | 3916 |
| Scaffold_14 | 318 | 1138 | 648 | 204 | 846 | 32 | 56 | 1786 |
| Scaffold_15 | 275 | 1031 | 530 | 212 | 726 | 25 | 68 | 1561 |
| Scaffold_16 | 353 | 1251 | 1226 | 226 | 913 | 39 | 73 | 2477 |
| **Total** | **8879** | **35347** | **28722** | **6712** | **25724** | **1034** | **1877** | **64069** |

**Supplementary Table 3.** Single-nucleotide polymorphism statistics and frequency of allele occurrence in the 64k SNP chip, where (A:G) indicate that A is a reference allele and G is the alternate allele.

| **S.No.** | **Alleles** | **Allele Occurrence** | **Percentage frequency of allele occurrence** |
| --- | --- | --- | --- |
| 1 | A:G | 9048 | 14.12 |
| 2 | T:C | 8445 | 13.18 |
| 3 | C:T | 7094 | 11.07 |
| 4 | G:A | 7038 | 10.99 |
| 5 | T:A | 4102 | 6.40 |
| 6 | A:T | 3502 | 5.47 |
| 7 | A:A | 3065 | 4.78 |
| 8 | C:C | 2929 | 4.57 |
| 9 | T:T | 2896 | 4.52 |
| 10 | G:G | 2786 | 4.35 |
| 11 | A:C | 2729 | 4.26 |
| 12 | T:G | 2553 | 3.98 |
| 13 | C:A | 2118 | 3.31 |
| 14 | G:T | 2066 | 3.22 |
| 15 | G:C | 1892 | 2.95 |
| 16 | C:G | 1806 | 2.82 |

**Supplementary Table 4.** Comparative list of genetic diversity indices estimated for the total collection and core sets.

| **Parameters** | **Total Collection (917 accessions)** | **Core Set (112 accessions)** |
| --- | --- | --- |
| GD | 0.1-0.5 (0.23) | 0.04-0.5 (0.25) |
| PIC | 0.09-0.38 (0.2) | 0.04-0.38 (0.21) |
| MAF | 0.05-0.5 (0.15) | 0.02-0.5 (0.16) |
| Ho | 0.04-0.62 (0.11) | 0.05-0.62 (0.11) |

**Supplementary Table 5.** List of 112 Amaranth core set accessions.

| **S.no.** | **Accession No.** | **States** |
| --- | --- | --- |
| 1 | EC0519542 | exotic |
| 2 | EC0169615 | exotic |
| 3 | IC0038579 | himachal pradesh |
| 4 | IC0035434 | maharashtra |
| 5 | IC0554108 | uttarakhand |
| 6 | IC0554158 | uttarakhand |
| 7 | IC0095368 | himachal pradesh |
| 8 | IC0035566 | gujarat |
| 9 | IC0017926 | himachal pradesh |
| 10 | IC0469241 | himachal pradesh |
| 11 | IC0448675 | uttarakhand |
| 12 | IC0035476 | maharashtra |
| 13 | IC0038367 | himachal pradesh |
| 14 | IC0035487 | maharashtra |
| 15 | IC0120606 | others |
| 16 | IC0035673 | himachal pradesh |
| 17 | IC0554053 | uttarakhand |
| 18 | EC0519547 | exotic |
| 19 | IC0042316-1 | uttarakhand |
| 20 | IC0550693 | maharashtra |
| 21 | IC0094658 | himachal pradesh |
| 22 | IC0042258-11 | uttar pradesh |
| 23 | IC0553974 | uttarakhand |
| 24 | IC0095365 | himachal pradesh |
| 25 | IC0042315-8 | uttar pradesh |
| 26 | IC0095464 | tamil nadu |
| 27 | IC0021802-A | madhya pradesh |
| 28 | IC0095642 | madhya pradesh |
| 29 | IC0038048 | himachal pradesh |
| 30 | IC0107276 | himachal pradesh |
| 31 | IC0038190 | himachal pradesh |
| 32 | IC038229 | himachal pradesh |
| 33 | IC0415268 | himachal pradesh |
| 34 | IC0394084 | gujarat |
| 35 | IC0394111 | gujarat |
| 36 | IC0258400 | himachal pradesh |
| 37 | IC0120694 | others |
| 38 | IC0035536 | gujarat |
| 39 | IC038157 | himachal pradesh |
| 40 | IC0038628 | himachal pradesh |
| 41 | IC0415262 | himachal pradesh |
| 42 | IC0038595 | himachal pradesh |
| 43 | IC0047438 | uttar pradesh |
| 44 | IC0095631 | himachal pradesh |
| 45 | IC0448717 | uttarakhand |
| 46 | IC0035664 | himachal pradesh |
| 47 | IC0038226 | himachal pradesh |
| 48 | IC0081702 | uttarakhand |
| 49 | IC0415250 | himachal pradesh |
| 50 | IC0037323 | sikkim |
| 51 | IC0021799 | chattisgarh |
| 52 | IC0553981 | uttarakhand |
| 53 | IC0553952 | uttarakhand |
| 54 | IC0107283 | himachal pradesh |
| 55 | IC0038660 | himachal pradesh |
| 56 | IC0095476 | others |
| 57 | IC0554152 | uttarakhand |
| 58 | IC0095537 | others |
| 59 | IC0553998 | uttarakhand |
| 60 | IC0042002 | madhya pradesh |
| 61 | IC0035539 | gujarat |
| 62 | IC0448769 | uttarakhand |
| 63 | IC0554038 | uttarakhand |
| 64 | IC0448628 | uttarakhand |
| 65 | IC0448627 | uttarakhand |
| 66 | IC0035503 | maharashtra |
| 67 | IC0035675 | himachal pradesh |
| 68 | IC0329587 | himachal pradesh |
| 69 | IC0107233 | himachal pradesh |
| 70 | IC0035579 | gujarat |
| 71 | EC0519524 | exotic |
| 72 | IC0448751 | uttarakhand |
| 73 | IC0095459 | tamil nadu |
| 74 | IC0035698 | himachal pradesh |
| 75 | IC0107531 | himachal pradesh |
| 76 | IC0095433 | uttarakhand |
| 77 | IC0448633 | uttarakhand |
| 78 | IC0448729 | uttarakhand |
| 79 | IC0035425 | maharashtra |
| 80 | IC0394095 | gujarat |
| 81 | IC0038455 | himachal pradesh |
| 82 | IC0554062 | uttarakhand |
| 83 | IC0038183 | himachal pradesh |
| 84 | IC0038275 | himachal pradesh |
| 85 | IC0554080 | uttarakhand |
| 86 | IC0095288 | himachal pradesh |
| 87 | IC0038309 | himachal pradesh |
| 88 | EC0524458 | exotic |
| 89 | IC038374 | himachal pradesh |
| 90 | IC0038145 | himachal pradesh |
| 91 | IC0554103 | uttarakhand |
| 92 | IC0095608 | uttar pradesh |
| 93 | EC0146547 | exotic |
| 94 | IC0274472 | himachal pradesh |
| 95 | IC0394102 | gujarat |
| 96 | IC0448666 | uttarakhand |
| 97 | IC0108428 | himachal pradesh |
| 98 | IC0120573 | others |
| 99 | IC0038474 | himachal pradesh |
| 100 | IC0554148 | uttarakhand |
| 101 | IC0281925 | odisha |
| 102 | IC0448747 | uttarakhand |
| 103 | IC0105047 | himachal pradesh |
| 104 | IC0095600 | himachal pradesh |
| 105 | IC0038591 | himachal pradesh |
| 106 | IC0415220 | himachal pradesh |
| 107 | IC0042254-15 | uttarakhand |
| 108 | IC0035659 | gujarat |
| 109 | IC0120640 | others |
| 110 | IC0095382 | himachal pradesh |
| 111 | Annapurna | others |
| 112 | Suvarna | others |

**Supplementary Table 6.** List of allele frequency of total collection and core collection.

| **Alleles** | **Total collection(917 accessions)** | **Core set (112 accessions)** |
| --- | --- | --- |
| AA | 27.52 | 27.25 |
| CC | 17.69 | 17.739 |
| GG | 17.9 | 17.937 |
| TT | 26.329 | 26.095 |
| CT/TC | 2.6 | 2.74 |
| AG/GA | 3.036 | 3.103 |
| CG/GC | 2.427 | 2.544 |
| GT/TG | 0.766 | 0.807 |
| AT/TA | 0.987 | 1 |
| AC/CA | 0.746 | 0.784 |

**Supplementary Table 7.** List of 540 grain amaranth accessions used for GWAS study.

| **S. No.** | **Accessions** | **States** |
| --- | --- | --- |
| 1 | IC0038265 | Himachal Pradesh |
| 2 | IC0042345 | uttarakhand |
| 3 | IC0021925 | Madhya Pradesh |
| 4 | IC0547387 | Himachal Pradesh |
| 5 | IC0258400 | Himachal Pradesh |
| 6 | IC0553987 | uttarakhand |
| 7 | IC0553976 | uttarakhand |
| 8 | IC0274450 | Himachal Pradesh |
| 9 | IC0313273 | Himachal Pradesh |
| 10 | IC0279973 | Himachal Pradesh |
| 11 | IC0279968 | Himachal Pradesh |
| 12 | IC0467899 | Himachal Pradesh |
| 13 | IC0329513 | Himachal Pradesh |
| 14 | IC0329514 | Himachal Pradesh |
| 15 | IC0107569 | Himachal Pradesh |
| 16 | IC0107847 | Himachal Pradesh |
| 17 | IC0107301 | Himachal Pradesh |
| 18 | IC0120599 | others |
| 19 | IC0107848 | Himachal Pradesh |
| 20 | IC0038105 | Himachal Pradesh |
| 21 | IC0038559 | Himachal Pradesh |
| 22 | IC0038611 | Himachal Pradesh |
| 23 | IC0038560 | Himachal Pradesh |
| 24 | IC0038570 | Himachal Pradesh |
| 25 | IC0038576 | Himachal Pradesh |
| 26 | IC0095338 | Himachal Pradesh |
| 27 | IC0035562 | gujarat |
| 28 | IC0038231 | Himachal Pradesh |
| 29 | IC0554010 | uttarakhand |
| 30 | IC0554173 | uttarakhand |
| 31 | IC0448642 | uttarakhand |
| 32 | IC415592 | rajasthan |
| 33 | IC0035518 | gujarat |
| 34 | IC0035560 | gujarat |
| 35 | IC0021923 | chattisgarh |
| 36 | IC0107578 | Himachal Pradesh |
| 37 | IC0038328 | Himachal Pradesh |
| 38 | IC0038297 | Himachal Pradesh |
| 39 | IC0329492 | Himachal Pradesh |
| 40 | IC0107144 | Himachal Pradesh |
| 41 | EC0519550 | exotic |
| 42 | IC038366 | Himachal Pradesh |
| 43 | EC0377618 | exotic |
| 44 | IC0448701 | uttarakhand |
| 45 | IC0108431 | Himachal Pradesh |
| 46 | EC0387022 | exotic |
| 47 | IC0107840 | uttarakhand |
| 48 | IC0554053 | uttarakhand |
| 49 | IC0035489 | maharashtra |
| 50 | IC0035496 | maharashtra |
| 51 | IC0017933 | Himachal Pradesh |
| 52 | IC0017957 | Himachal Pradesh |
| 53 | IC0037320 | sikkim |
| 54 | IC0035696 | Himachal Pradesh |
| 55 | IC0107531 | Himachal Pradesh |
| 56 | IC0107276 | Himachal Pradesh |
| 57 | EC0387002 | exotic |
| 58 | IC0448786 | uttarakhand |
| 59 | IC0448791 | uttarakhand |
| 60 | IC0448788 | uttarakhand |
| 61 | IC0107838 | Himachal Pradesh |
| 62 | IC0506493 | Himachal Pradesh |
| 63 | IC0107829 | Himachal Pradesh |
| 64 | IC0107615 | Himachal Pradesh |
| 65 | IC0107617 | Himachal Pradesh |
| 66 | IC0107256 | Himachal Pradesh |
| 67 | IC0448758 | uttarakhand |
| 68 | IC0448724 | uttarakhand |
| 69 | IC0448735 | uttarakhand |
| 70 | IC0448740 | uttarakhand |
| 71 | IC0448716 | uttarakhand |
| 72 | IC0448720 | uttarakhand |
| 73 | IC0035553 | gujarat |
| 74 | IC0035670 | Himachal Pradesh |
| 75 | IC0448665 | uttarakhand |
| 76 | IC0448700 | uttarakhand |
| 77 | IC0448693 | uttarakhand |
| 78 | IC0035592 | gujarat |
| 79 | IC0035593 | gujarat |
| 80 | IC0035646 | gujarat |
| 81 | EC0359436 | exotic |
| 82 | IC0038110 | Himachal Pradesh |
| 83 | IC0042295 | uttarakhand |
| 84 | IC0038620 | Himachal Pradesh |
| 85 | IC0038574 | Himachal Pradesh |
| 86 | IC0095379 | Himachal Pradesh |
| 87 | IC0038647 | Himachal Pradesh |
| 88 | IC0038359 | Himachal Pradesh |
| 89 | IC0554158 | uttarakhand |
| 90 | IC0554108 | uttarakhand |
| 91 | IC0448792 | uttarakhand |
| 92 | IC0448772 | uttarakhand |
| 93 | IC0448783 | uttarakhand |
| 94 | IC0042258 | uttarpradesh |
| 95 | IC0448747 | uttarakhand |
| 96 | IC0278919 | Himachal Pradesh |
| 97 | IC0038150 | Himachal Pradesh |
| 98 | IC0038129 | Himachal Pradesh |
| 99 | IC0042254-14 | uttarakhand |
| 100 | IC038510 | Himachal Pradesh |
| 101 | IC038229 | Himachal Pradesh |
| 102 | IC0313263 | Himachal Pradesh |
| 103 | IC0042006 | Madhya Pradesh |
| 104 | IC0038276 | Himachal Pradesh |
| 105 | IC0035511 | gujarat |
| 106 | IC0038365 | Himachal Pradesh |
| 107 | IC0042090-17 | others |
| 108 | IC0038657 | Himachal Pradesh |
| 109 | IC0313274 | Himachal Pradesh |
| 110 | IC0038097 | Himachal Pradesh |
| 111 | IC0038058 | Himachal Pradesh |
| 112 | IC0469235 | Himachal Pradesh |
| 113 | IC0469242 | Himachal Pradesh |
| 114 | IC0506602 | uttarakhand |
| 115 | IC0042290-20 | uttarakhand |
| 116 | EC0519542 | exotic |
| 117 | IC0035409 | maharashtra |
| 118 | IC0553995 | uttarakhand |
| 119 | IC0038542 | Himachal Pradesh |
| 120 | IC0038443 | Himachal Pradesh |
| 121 | IC0047434 | chattisgarh |
| 122 | EC0359435 | exotic |
| 123 | IC0042281-11 | uttarakhand |
| 124 | IC0038133 | Himachal Pradesh |
| 125 | IC0038103 | Himachal Pradesh |
| 126 | IC0035551 | gujarat |
| 127 | IC0042267-4 | uttarakhand |
| 128 | IC0035611 | gujarat |
| 129 | IC0035590 | gujarat |
| 130 | IC0415250 | Himachal Pradesh |
| 131 | IC0038390 | Himachal Pradesh |
| 132 | IC0035623 | gujarat |
| 133 | EC0198135 | exotic |
| 134 | IC0107696 | Himachal Pradesh |
| 135 | IC0035478 | maharashtra |
| 136 | IC0021789 | Madhya Pradesh |
| 137 | IC0095503 | uttarpradesh |
| 138 | IC0553983 | uttarakhand |
| 139 | IC0553973 | uttarakhand |
| 140 | IC0554128 | uttarakhand |
| 141 | IC0554116 | uttarakhand |
| 142 | IC0035541 | gujarat |
| 143 | IC0120643 | others |
| 144 | IC0120655 | others |
| 145 | IC0042258-13 | uttarpradesh |
| 146 | IC0042254-15 | uttarakhand |
| 147 | IC0038445 | Himachal Pradesh |
| 148 | IC0035552 | gujarat |
| 149 | IC0035393 | maharashtra |
| 150 | IC0038132 | Himachal Pradesh |
| 151 | IC0042002 | Madhya Pradesh |
| 152 | IC0038534 | gujarat |
| 153 | IC0038250 | Himachal Pradesh |
| 154 | IC0506595 | others |
| 155 | EC0169625 | exotic |
| 156 | IC0618539 | nagaland |
| 157 | IC0038171 | Himachal Pradesh |
| 158 | IC0035640 | gujarat |
| 159 | IC0035566 | gujarat |
| 160 | IC0095533 | others |
| 161 | IC0038286 | Himachal Pradesh |
| 162 | IC0035386 | maharashtra |
| 163 | IC0448741 | uttarakhand |
| 164 | IC0021926 | chattisgarh |
| 165 | IC0005626 | others |
| 166 | IC0018369 | Himachal Pradesh |
| 167 | IC0035539 | gujarat |
| 168 | EC0387012 | exotic |
| 169 | IC0448769 | uttarakhand |
| 170 | IC0448787 | uttarakhand |
| 171 | IC0448725 | uttarakhand |
| 172 | IC0448719 | uttarakhand |
| 173 | IC0448749 | uttarakhand |
| 174 | IC0448723 | uttarakhand |
| 175 | IC0095453 | kerala |
| 176 | IC0042258-10 | uttarpradesh |
| 177 | IC0274462 | Himachal Pradesh |
| 178 | IC0038102 | Himachal Pradesh |
| 179 | IC0038226 | Himachal Pradesh |
| 180 | IC0038368 | Himachal Pradesh |
| 181 | IC0415318 | Himachal Pradesh |
| 182 | EC0524458 | exotic |
| 183 | IC0095380 | Himachal Pradesh |
| 184 | IC0444147 | uttarakhand |
| 185 | IC0553998 | uttarakhand |
| 186 | IC0120568 | others |
| 187 | IC0278912 | Himachal Pradesh |
| 188 | IC0313271 | Himachal Pradesh |
| 189 | IC0042264-X | uttarpradesh |
| 190 | IC0469241 | Himachal Pradesh |
| 191 | IC0095590 | Himachal Pradesh |
| 192 | IC0042315-3 | uttarpradesh |
| 193 | EC0169611 | exotic |
| 194 | IC0094652 | andhrapradesh |
| 195 | IC0094658 | Himachal Pradesh |
| 196 | IC0035766 | Himachal Pradesh |
| 197 | IC0038505 | Himachal Pradesh |
| 198 | IC0038540 | Himachal Pradesh |
| 199 | IC0038483 | Himachal Pradesh |
| 200 | IC0038545 | Himachal Pradesh |
| 201 | IC0038482 | Himachal Pradesh |
| 202 | IC0038536 | Himachal Pradesh |
| 203 | IC0095337 | Himachal Pradesh |
| 204 | IC0035564 | gujarat |
| 205 | IC0042279-2 | uttarakhand |
| 206 | IC0042277-9 | uttarakhand |
| 207 | IC0042284-17 | uttarakhand |
| 208 | IC038175 | Himachal Pradesh |
| 209 | IC038369 | Himachal Pradesh |
| 210 | IC0415254 | Himachal Pradesh |
| 211 | IC0038528 | Himachal Pradesh |
| 212 | IC415591 | rajasthan |
| 213 | IC0095256 | Himachal Pradesh |
| 214 | IC0035669 | Himachal Pradesh |
| 215 | IC0035543 | gujarat |
| 216 | IC0042279-6 | uttarakhand |
| 217 | IC0038464 | Himachal Pradesh |
| 218 | IC0035414 | maharashtra |
| 219 | IC0035436 | maharashtra |
| 220 | IC0107688 | Himachal Pradesh |
| 221 | IC0554079 | uttarakhand |
| 222 | IC0554091 | uttarakhand |
| 223 | IC0035671 | Himachal Pradesh |
| 224 | IC0554078 | uttarakhand |
| 225 | IC0553952 | uttarakhand |
| 226 | IC0553981 | uttarakhand |
| 227 | IC0448689 | uttarakhand |
| 228 | IC0448694 | uttarakhand |
| 229 | IC0448618 | uttarakhand |
| 230 | IC0035582 | gujarat |
| 231 | IC0035634 | gujarat |
| 232 | IC0035675 | Himachal Pradesh |
| 233 | IC0035557 | gujarat |
| 234 | IC0035638 | gujarat |
| 235 | IC0037323 | sikkim |
| 236 | IC0035607 | gujarat |
| 237 | EC0169622 | exotic |
| 238 | IC0553974 | uttarakhand |
| 239 | IC0038052 | Himachal Pradesh |
| 240 | IC0042264 | uttarpradesh |
| 241 | IC0448635 | uttarakhand |
| 242 | IC0448658 | uttarakhand |
| 243 | IC0547375 | Himachal Pradesh |
| 244 | IC0035530 | gujarat |
| 245 | IC0038635 | Himachal Pradesh |
| 246 | IC0038633 | Himachal Pradesh |
| 247 | IC0038367 | Himachal Pradesh |
| 248 | IC0554126 | uttarakhand |
| 249 | IC0448759 | uttarakhand |
| 250 | IC0448734 | uttarakhand |
| 251 | IC0448761 | uttarakhand |
| 252 | IC038392 | Himachal Pradesh |
| 253 | IC0007930 | others |
| 254 | IC0038299 | Himachal Pradesh |
| 255 | IC0017926 | Himachal Pradesh |
| 256 | IC0025788 | arunachalpradesh |
| 257 | IC0467884 | Himachal Pradesh |
| 258 | EC0120050 | exotic |
| 259 | IC0041999 | Madhya Pradesh |
| 260 | IC0017940 | Himachal Pradesh |
| 261 | IC0035450 | maharashtra |
| 262 | IC0554056 | uttarakhand |
| 263 | IC0554067 | uttarakhand |
| 264 | IC0554073 | uttarakhand |
| 265 | IC0554065 | uttarakhand |
| 266 | IC0553988 | uttarakhand |
| 267 | IC0038431 | Himachal Pradesh |
| 268 | IC0038257 | Himachal Pradesh |
| 269 | IC0038335 | Himachal Pradesh |
| 270 | IC0553982 | uttarakhand |
| 271 | IC0310049 | Himachal Pradesh |
| 272 | IC0361603 | Himachal Pradesh |
| 273 | IC0361608 | Himachal Pradesh |
| 274 | IC0042301 | uttarpradesh |
| 275 | IC0035706 | Himachal Pradesh |
| 276 | IC0038099 | Himachal Pradesh |
| 277 | IC0423410 | Himachal Pradesh |
| 278 | IC0415268 | Himachal Pradesh |
| 279 | IC0038579 | Himachal Pradesh |
| 280 | IC0038577-3 | Himachal Pradesh |
| 281 | IC0035616 | gujarat |
| 282 | IC0035658 | gujarat |
| 283 | IC0035619 | gujarat |
| 284 | IC0035618 | gujarat |
| 285 | IC0038085 | Himachal Pradesh |
| 286 | IC0038064 | Himachal Pradesh |
| 287 | IC0105047 | Himachal Pradesh |
| 288 | IC0106354 | Himachal Pradesh |
| 289 | EC0223642 | exotic |
| 290 | IC0243176 | Himachal Pradesh |
| 291 | IC0423537 | Himachal Pradesh |
| 292 | IC0553959 | uttarakhand |
| 293 | IC0554002 | uttarakhand |
| 294 | IC0553954 | uttarakhand |
| 295 | IC0553961 | uttarakhand |
| 296 | IC0093942 | others |
| 297 | IC0095556 | maharashtra |
| 298 | IC0038240 | Himachal Pradesh |
| 299 | IC0038487 | Himachal Pradesh |
| 300 | IC0095321 | Himachal Pradesh |
| 301 | IC0107835 | Himachal Pradesh |
| 302 | IC0108429 | Himachal Pradesh |
| 303 | IC0042264-6 | uttarpradesh |
| 304 | IC0042264-14 | uttarpradesh |
| 305 | IC038159 | Himachal Pradesh |
| 306 | IC0035548 | gujarat |
| 307 | EC0519547 | exotic |
| 308 | IC0035441 | maharashtra |
| 309 | IC0110271 | others |
| 310 | IC0095433 | uttarakhand |
| 311 | IC0095318 | others |
| 312 | IC0021802 | Madhya Pradesh |
| 313 | IC0035580 | Himachal Pradesh |
| 314 | IC0038639 | Himachal Pradesh |
| 315 | IC0038614 | Himachal Pradesh |
| 316 | IC0038621 | Himachal Pradesh |
| 317 | IC0038645 | Himachal Pradesh |
| 318 | IC0095352 | Himachal Pradesh |
| 319 | IC0094659 | Himachal Pradesh |
| 320 | IC0016638 | uttarakhand |
| 321 | IC0038046 | haryana |
| 322 | IC0038041 | uttarpradesh |
| 323 | IC0042275-2 | uttarakhand |
| 324 | IC0042292-11 | uttarakhand |
| 325 | IC0095204 | others |
| 326 | IC0095363 | Himachal Pradesh |
| 327 | IC0095598 | Himachal Pradesh |
| 328 | IC0035373 | maharashtra |
| 329 | IC0035401 | maharashtra |
| 330 | IC0035741 | Himachal Pradesh |
| 331 | IC0035686 | Himachal Pradesh |
| 332 | IC0035689 | Himachal Pradesh |
| 333 | IC0095248 | Himachal Pradesh |
| 334 | IC0095283 | Himachal Pradesh |
| 335 | IC0081710 | uttarakhand |
| 336 | IC0038127 | Himachal Pradesh |
| 337 | IC0506505 | Himachal Pradesh |
| 338 | IC0107291 | Himachal Pradesh |
| 339 | IC0554038 | uttarakhand |
| 340 | IC0554017 | uttarakhand |
| 341 | IC0448726 | uttarakhand |
| 342 | IC0448763 | uttarakhand |
| 343 | IC0448750 | uttarakhand |
| 344 | IC0448675 | uttarakhand |
| 345 | IC0448696 | uttarakhand |
| 346 | IC0448646 | uttarakhand |
| 347 | IC0448702 | uttarakhand |
| 348 | IC0448666 | uttarakhand |
| 349 | IC0448713 | uttarakhand |
| 350 | IC0035558 | gujarat |
| 351 | EC0322994 | exotic |
| 352 | IC0554152 | uttarakhand |
| 353 | IC0554150 | uttarakhand |
| 354 | IC0554101 | uttarakhand |
| 355 | IC0554120 | uttarakhand |
| 356 | IC0042277 | uttarakhand |
| 357 | IC0095459 | tamilnadu |
| 358 | IC0021927 | chattisgarh |
| 359 | IC0038610 | Himachal Pradesh |
| 360 | IC0055149 | maharashtra |
| 361 | IC0038643 | Himachal Pradesh |
| 362 | IC0095464 | tamilnadu |
| 363 | IC0095466 | tamilnadu |
| 364 | IC0095475 | others |
| 365 | IC0018366 | Himachal Pradesh |
| 366 | IC0042261-1 | uttarpradesh |
| 367 | IC0042258-11 | uttarpradesh |
| 368 | IC0042258-1 | uttarpradesh |
| 369 | IC0007941 | others |
| 370 | IC0035487 | maharashtra |
| 371 | IC0038302 | Himachal Pradesh |
| 372 | IC0035721 | Himachal Pradesh |
| 373 | IC0035694 | Himachal Pradesh |
| 374 | IC0035731 | Himachal Pradesh |
| 375 | IC0035780 | gujarat |
| 376 | IC0041988 | Madhya Pradesh |
| 377 | IC0095367 | Himachal Pradesh |
| 378 | IC0095368 | Himachal Pradesh |
| 379 | IC-55143 | maharashtra |
| 380 | IC0095581 | Himachal Pradesh |
| 381 | IC0095601 | uttarpradesh |
| 382 | IC0095643 | maharashtra |
| 383 | IC0554069 | uttarakhand |
| 384 | IC0553963 | uttarakhand |
| 385 | IC0120603 | others |
| 386 | IC0120606 | others |
| 387 | IC0095562 | Himachal Pradesh |
| 388 | IC0042358 | uttarakhand |
| 389 | IC0042315-5 | uttarpradesh |
| 390 | IC0042293-16 | uttarakhand |
| 391 | IC0042293-8 | uttarakhand |
| 392 | IC0042290-16 | uttarakhand |
| 393 | IC0095394 | Himachal Pradesh |
| 394 | IC0095346 | Himachal Pradesh |
| 395 | IC0095642 | Madhya Pradesh |
| 396 | IC0038517 | Himachal Pradesh |
| 397 | IC0038455 | Himachal Pradesh |
| 398 | IC0108428 | Himachal Pradesh |
| 399 | IC0035774 | gujarat |
| 400 | IC0042283-8 | uttarakhand |
| 401 | IC0042267 | uttarakhand |
| 402 | IC038170 | Himachal Pradesh |
| 403 | EC0519540 | exotic |
| 404 | IC0035736 | Himachal Pradesh |
| 405 | IC0095277 | Himachal Pradesh |
| 406 | IC0412770 | arunachalpradesh |
| 407 | IC0038048 | Himachal Pradesh |
| 408 | IC0095449 | tamilnadu |
| 409 | IC0095483 | others |
| 410 | EC0169606 | exotic |
| 411 | IC0448755 | uttarakhand |
| 412 | IC0017950 | Himachal Pradesh |
| 413 | IC0554066 | uttarakhand |
| 414 | IC0038200 | Himachal Pradesh |
| 415 | IC0035420 | maharashtra |
| 416 | IC0038575 | Himachal Pradesh |
| 417 | IC0035434 | maharashtra |
| 418 | IC0038190 | Himachal Pradesh |
| 419 | IC0554163 | uttarakhand |
| 420 | IC0042332 | uttarpradesh |
| 421 | IC0042316-11 | uttarpradesh |
| 422 | EC0169615 | exotic |
| 423 | IC038374 | Himachal Pradesh |
| 424 | IC038166 | Himachal Pradesh |
| 425 | IC0528306 | uttarpradesh |
| 426 | IC0095582 | Himachal Pradesh |
| 427 | IC0035683 | Himachal Pradesh |
| 428 | IC0095288 | Himachal Pradesh |
| 429 | IC0095305 | others |
| 430 | IC0095489 | others |
| 431 | IC0095463 | tamilnadu |
| 432 | IC0120653 | others |
| 433 | IC0035673 | Himachal Pradesh |
| 434 | IC0625391 | nagaland |
| 435 | IC0095617 | others |
| 436 | IC0035685 | Himachal Pradesh |
| 437 | IC0095479 | maharashtra |
| 438 | IC0120569 | others |
| 439 | IC0035656 | gujarat |
| 440 | IC0095542 | uttarakhand |
| 441 | IC0095365 | Himachal Pradesh |
| 442 | IC0095386 | Himachal Pradesh |
| 443 | IC0021796-A | chattisgarh |
| 444 | IC0035485 | maharashtra |
| 445 | IC0035412 | maharashtra |
| 446 | IC0095259 | others |
| 447 | IC0095382 | Himachal Pradesh |
| 448 | IC0095389 | Himachal Pradesh |
| 449 | IC0095450 | tamilnadu |
| 450 | IC0081702 | uttarakhand |
| 451 | IC0095257 | others |
| 452 | IC0035702 | Himachal Pradesh |
| 453 | IC0035534 | gujarat |
| 454 | EC0359431 | exotic |
| 455 | IC0021966 | chattisgarh |
| 456 | IC0035503 | maharashtra |
| 457 | IC0035394 | maharashtra |
| 458 | IC0554161 | uttarakhand |
| 459 | IC0627440 | rajasthan |
| 460 | IC0107007 | Himachal Pradesh |
| 461 | IC0038205 | Himachal Pradesh |
| 462 | IC0448641 | uttarakhand |
| 463 | IC0448655 | uttarakhand |
| 464 | IC0035431 | maharashtra |
| 465 | IC0095537 | others |
| 466 | IC0554165 | uttarakhand |
| 467 | IC0554168 | uttarakhand |
| 468 | IC0554137 | uttarakhand |
| 469 | IC0249597 | telangana |
| 470 | IC0095474 | others |
| 471 | IC0035445 | maharashtra |
| 472 | IC0095465 | tamilnadu |
| 473 | IC0095455 | tamilnadu |
| 474 | IC0281942 | odisha |
| 475 | IC0281933 | odisha |
| 476 | IC0281925 | odisha |
| 477 | IC0021808 | Madhya Pradesh |
| 478 | IC0021944 | Madhya Pradesh |
| 479 | IC0042011 | maharashtra |
| 480 | IC0095245 | Himachal Pradesh |
| 481 | IC0038395 | Himachal Pradesh |
| 482 | IC-35791 | others |
| 483 | IC0035449 | maharashtra |
| 484 | IC0394085 | gujarat |
| 485 | IC0120693 | others |
| 486 | IC0120613 | others |
| 487 | IC0095541 | uttarpradesh |
| 488 | IC0120696 | others |
| 489 | IC0095600 | Himachal Pradesh |
| 490 | IC0398215 | gujarat |
| 491 | IC0038543 | Himachal Pradesh |
| 492 | IC0095247 | Himachal Pradesh |
| 493 | IC0042315-8 | uttarpradesh |
| 494 | IC0042277-2 | uttarakhand |
| 495 | IC0394091 | gujarat |
| 496 | IC0394092 | gujarat |
| 497 | IC0394087 | gujarat |
| 498 | IC0394095 | gujarat |
| 499 | IC0035438 | maharashtra |
| 500 | IC0038524 | Himachal Pradesh |
| 501 | IC469674 | Karnataka |
| 502 | IC0041992 | Madhya Pradesh |
| 503 | IC0110269 | others |
| 504 | IC0095566 | Himachal Pradesh |
| 505 | IC0035506 | maharashtra |
| 506 | IC0038187 | kerala |
| 507 | IC0035370 | maharashtra |
| 508 | IC0035362 | maharashtra |
| 509 | EC0198117 | exotic |
| 510 | EC0198136 | exotic |
| 511 | IC0038212 | Himachal Pradesh |
| 512 | IC0017954 | Himachal Pradesh |
| 513 | IC0095610 | others |
| 514 | IC0095531 | maharashtra |
| 515 | IC0120567 | others |
| 516 | IC0038275 | Himachal Pradesh |
| 517 | IC0035407 | maharashtra |
| 518 | IC0035659 | gujarat |
| 519 | IC0107334 | Himachal Pradesh |
| 520 | IC0095544 | uttarakhand |
| 521 | IC0038202 | Himachal Pradesh |
| 522 | IC0038124 | Himachal Pradesh |
| 523 | IC0035476 | maharashtra |
| 524 | IC0095282 | Himachal Pradesh |
| 525 | IC0035614 | gujarat |
| 526 | IC0035403 | maharashtra |
| 527 | IC0032179 | maharashtra |
| 528 | IC0021802-A | Madhya Pradesh |
| 529 | IC0035444 | maharashtra |
| 530 | IC0035366 | maharashtra |
| 531 | IC0035465 | maharashtra |
| 532 | IC0120694 | others |
| 533 | IC0120691 | others |
| 534 | IC0618379 | maharashtra |
| 535 | IC0035372 | maharashtra |
| 536 | IC0281919 | odisha |
| 537 | IC0281921 | odisha |
| 538 | IC0120676 | others |
| 539 | IC0038164 | Himachal Pradesh |
| 540 | IC0038072 | Himachal Pradesh |

**Supplementary Table 8.** List of significant QTNs for DTF traits detected simultaneously using SL-GWAS and ML-GWAS methods in two environments, E1 and E2.

| **Trait** | **Environment** | **QTN** | **Scaffold** | **Position (bp)** | **LOD score** | **r2 (%)** | **QTN effect** | **Method** | **Linked Gene** |
| --- | --- | --- | --- | --- | --- | --- | --- | --- | --- |
| Days to flowering | E1 | *qDTF-1-1* | 1 | 3320367 | 5.5011 | 6.0177 | -3.8378 | 3,4 |  |
|  |  | *qDTF-1-2* | 1 | 29118797 | 3.5459 | 5.1596 | 2.4596 | 3,6 |  |
|  |  | *qDTF-1-3* | 1 | 27292090 | 3.2348 | 5.2778 | -2.8312 | 3,6 |  |
|  |  | *qDTF-4-1* | 4 | 7377624 | 3.1843 | 2.2995 | 1.677 | 1,2,3,5,6 |  |
|  |  | *qDTF-4-2* | 4 | 20352653 | 3.5245 | 4.5271 | 3.1303 | 1,2,5 | AH007573 |
|  |  | *qDTF-5-1* | 5 | 2338055 | 5.2849 | 2.6664 | 4.702 | 1,2,4 | AH008526 |
|  |  | *qDTF-7-1* | 7 | 16342396 | 4.3828 | 3.1632 | -2.6174 | 1,2,3,4,6 | AH011805 |
|  |  | *qDTF-7-2* | 7 | 21785289 | 4.7717 | 3.6541 | -3.0278 | 1,2,3,4,5,6 |  |
|  |  | *qDTF-8-1* | 8 | 11128790 | 3.6066 | 8.4002 | 4.0338 | 1,2,3,5 |  |
|  |  | *qDTF-14-1* | 14 | 19884801 | 3.8836 | 2.4156 | -1.6474 | 1,2,3,5,6 |  |
|  |  | *qDTF-16-1* | 16 | 1220745 | 3.7798 | 3.5278 | -2.854 | 3,6 |  |
|  | E2 | *qDTF-1-1* | 1 | 22191367 | 6.9316 | 10.7168 | -8.3313 | 1,2,4 | AH001195 |
|  |  | *qDTF-1-2* | 1 | 22247082 | 5.9151 | 4.7692 | -3.0499 | 1,5 |  |
|  |  | *qDTF-1-3* | 1 | 25268695 | 6.0889 | 12.9119 | -4.0975 | 3,5,6 |  |
|  |  | *qDTF-2-1* | 2 | 34857264 | 4.3683 | 5.0593 | 3.0684 | 1,2,3,5,6 |  |
|  |  | *qDTF-6-1* | 6 | 21991300 | 3.5356 | 3.8949 | 2.3817 | 3,6 | AH010969 |
|  |  | *qDTF-7-1* | 7 | 8796105 | 6.8773 | 5.2489 | -1.9758 | 3,6 |  |
|  |  | *qDTF-7-2* | 7 | 16342396 | 3.3788 | 1.717 | -3.3029 | 1,2,4,5,6 | AH011805 |
|  |  | *qDTF-9-1* | 9 | 12602621 | 5.4167 | 11.0933 | -4.7211 | 1,2,3,5,6 | AH014416 |
|  |  | *qDTF-10-1* | 10 | 22355768 | 6.6621 | 4.3431 | -1.7913 | 3,6 |  |
|  |  | *qDTF-13-1* | 13 | 13488267 | 6.2021 | 3.0587 | 2.6599 | 1,2,3 | AH019886 |
|  |  | *qDTF-13-2* | 13 | 19178528 | 3.9005 | 1.943 | 1.3484 | 3,6 |  |

**Supplementary Table 9. List of 54 potential candidate genes identified associated to DTF trait.**

| **Gene ID** | **Functional Annotation (Phytozome 13)** | **Functional Annotation (AGRDB)** |
| --- | --- | --- |
| AH007559 | PROTEIN-RELATED // SUBFAMILY NOT NAMED | F-box At1g55000 |
| AH007563 | PROTEIN ALWAYS EARLY 1-RELATED | ALWAYS EARLY 2-like isoform X1 |
| AH007572 | F-BOX/KELCH-REPEAT PROTEIN SKIP4-RELATED | F-box kelch-repeat SKIP4 |
| AH007573 | ZF-4CXXC-R1 TRANSCRIPTION FACTOR AND JUMONJI DOMAIN-CONTAINING PROTEIN | Lysine-specific demethylase JMJ25-like |
| AH007577 | Tudor/PWWP/MBT superfamily protein AT3G05430.1 | PWWP domain-containing |
| AH007579 | Tudor/PWWP/MBT superfamily protein AT3G05430.1 | PWWP domain containing |
| AH008516 | TUBBY-LIKE F-BOX PROTEIN 1-RELATED | Tubby-like F-box 8 |
| AH008519 | BASIC LEUCINE ZIPPER TRANSCRIPTION FACTOR-RELATED | G-box-binding factor 4-like |
| AH008524 | THIOREDOXIN Y1 | Thioredoxin chloroplastic-like |
| AH008526 | ABA/WDS induced protein (ABA_WDS) | Stress DDR48-like |
| AH008527 | ABA/WDS induced protein (ABA_WDS) | Abscisic stress-ripening 1-like |
| AH008528 | ABA/WDS induced protein (ABA_WDS) | Abscisic stress-ripening 3 isoform X2 |
| AH008529 | ABA/WDS induced protein (ABA_WDS) | ASR2 |
| AH008531 | Glycosylphosphatidylinositol transamidase (GAA1) | Glycosylphosphatidylinositol anchor attachment 1 |
| AH011791 | PPR repeat (PPR) // PPR repeat family (PPR_2) | Pentatricopeptide repeat-containing protein At3g06430, chloroplastic |
| AH011793 | ATP citrate (pro-S)-lyase (ACLY) | ATP-citrate synthase alpha chain protein 2 |
| AH011804 | ubiquitin-protein ligase E3 C (UBE3C) | E3 ubiquitin-protein ligase UPL6 |
| AH011805 | NUCLEAR EXPORT MEDIATOR FACTOR NEMF | Nuclear export mediator factor Nemf |
| AH011814 | AUXIN-RESPONSIVE PROTEIN IAA31 | Auxin-responsive protein IAA31 isoform X1 |
| AH011815 | AGAMOUS-LIKE MADS-BOX PROTEIN AGL36-RELATED | Agamous-like MADS-box protein AGL80 |
| AH011817 | MYB DOMAIN PROTEIN 78 | Transcription factor MYB108-like |
| AH011819 | de-etiolated-1 (DET1) | Light-mediated development protein DET1 isoform X1 |
| AH001181 | Plant protein of unknown function (DUF641) (DUF641) | GRAVITROPIC IN THE LIGHT 1 |
| AH001182 | Zinc finger CCCH domain-containing 16-like isoform X1 | Zinc finger CCCH domain-containing 16-like isoform X1 |
| AH001183 | Plant protein of unknown function (DUF641) (DUF641) | GRAVITROPIC IN THE LIGHT 1 |
| AH001194 | F-box and associated interaction domains-containing protein AT3G23880.1 | F-box kelch-repeat At3g23880-like |
| AH001195 | EXPRESSED PROTEIN | Uncharacterized membrane At3g27390 |
| AH001198 | F-box-like (F-box-like) | F-box SNE |
| AH014379 | ZINC FINGER PROTEIN CONSTANS-LIKE 5 | Zinc finger protein CONSTANS-LIKE 5 |
| AH014386 | MYB-LIKE DNA-BINDING PROTEIN MYB // SUBFAMILY | Transcription factor RAX3-like |
| AH014389 | C2H2 zinc finger protein, expressed LOC_Os03g13600.2 | Zinc finger protein 2 |
| AH014390 | 4-coumarate--CoA ligase / 4-coumaryl-CoA synthetase | 4-coumarate--CoA ligase 1-like |
| AH014397 | AUXIN-RESPONSIVE PROTEIN IAA33 | Auxin-responsive protein IAA33 |
| AH014400 | - | Homeobox protein |
| AH014401 | - | AP2-like ethylene-responsive transcription factor AIL5 |
| AH014402 | AP2-like ethylene-responsive transcription factor AIL5 | AP2-like ethylene-responsive transcription factor AIL5 |
| AH014416 | RIBOSOMAL PROTEIN L5-RELATED | 60S ribosomal protein L5 |
| AH019852 | ubiquitin-conjugating enzyme E2 J1 (UBE2J1 | Ubiquitin-conjugating enzyme E2 32 |
| AH019853 | Root cap and Late embryogenesis related family protein precursor, expressed LOC_Os05g47940.1 | Leucine-rich repeat extensin-like protein 3 |
| AH019857 | CAMP-RESPONSE ELEMENT BINDING PROTEIN-RELATED // SUBFAMILY NOT NAMED | BZIP transcription factor 53-like |
| AH019863 | CALMODULIN-BINDING TRANSCRIPTION ACTIVATOR 5-RELATED | Calmodulin-binding transcription activator 5 isoform X1 |
| AH019869 | FAR1 DNA-binding domain (FAR1) | Protein FAR1-RELATED SEQUENCE 11-like |
| AH019870 | ARMADILLO/BETA-CATENIN-LIKE REPEATS-CONTAINING PROTEIN-RELATED | Importin subunit alpha-1 |
| AH019871 | SWIM zinc finger (SWIM) | Protein FAR1-RELATED SEQUENCE 11-like |
| AH019872 | ARMADILLO/BETA-CATENIN-LIKE REPEATS-CONTAINING PROTEIN-RELATED | Importin subunit alpha-2-like |
| AH019873 | GATA TRANSCRIPTION FACTOR 15-RELATED | GATA transcription factor 15-like |
| AH019874 | GATA zinc finger (GATA) | GATA transcription factor 15-like |
| AH019875 | Tetratricopeptide repeat (TPR_1) | Protein KINESIN LIGHT CHAIN-RELATED 3-like |
| AH019886 | TLD-domain containing nucleolar protein AT2G05590.2 | Oxidation resistance protein 1-like |
| AH019896 | PLATZ transcription factor (PLATZ) | PLATZ transcription factor family protein |
| AH019899 | G-PROTEIN COUPLED RECEPTOR 89-RELATED | GPCR-type G protein 1 |
| AH019900 | QLQ (QLQ) | Growth-regulating factor 4-like |
| AH019916 | WD-REPEAT PROTEIN | WD repeat-containing protein DWA2-like |
| AH019919 | COP1-INTERACTING PROTEIN-LIKE PROTEIN | COP1-interacting protein 7 |
